# Report from the 27th (Virtual) Meeting on Toxinology, “Toxins: Mr Hyde or Dr Jekyll?”, Organized by the French Society of Toxinology, 9–10 December 2021

**DOI:** 10.3390/toxins14020110

**Published:** 2022-02-01

**Authors:** Daniel Ladant, Pascale Marchot, Sylvie Diochot, Gilles Prévost, Michel R. Popoff, Evelyne Benoit

**Affiliations:** 1 Institut Pasteur, Unité Biochimie des Interactions Macromoléculaires, 25-28 rue du Docteur Roux, 75015 Paris, France; daniel.ladant@pasteur.fr; 2 Laboratoire Architecture et Fonction des Macromolécules Biologiques, CNRS/Aix-Marseille Université, Faculté des Sciences—Campus Luminy, 13288 Marseille, France; pascale.marchot@univ-amu.fr; 3 Institut de Pharmacologie Moléculaire et Cellulaire, Université Côte d’Azur, CNRS, Sophia Antipolis, 06560 Valbonne, France; diochot@ipmc.cnrs.fr; 4 Institut de Bactériologie, Unité UR-7290 Virulence Bactérienne Précoce, ITI InnoVec, 3 rue Koeberlé, 67000 Strasbourg, France; prevost@unistra.fr; 5 Institut Pasteur, Unité Toxines Bactériennes, 25-28 rue du Docteur Roux, 75015 Paris, France; popoff2m@gmail.com; 6 CEA, Institut des Sciences du Vivant Frédéric Joliot, Département Médicaments et Technologies pour la Santé (DMTS), Service d’Ingénierie Moléculaire pour la Santé (SIMoS), Université Paris-Saclay, EMR 9004 CNRS/CEA, 91191 Gif-sur-Yvette, France

**Keywords:** animal toxin, bacterial toxin, marine toxin, medical application, plant toxin, toxin function/activity, toxin receptor/target, toxin structure

## Abstract

The French Society of Toxinology (SFET) organized its 27th annual meeting on 9–10 December 2021 as a virtual meeting (e-RT27). The central theme of this meeting was “Toxins: Mr Hyde or Dr Jekyll?”, emphasizing the latest findings on plant, fungal, algal, animal and bacterial toxins during 10 lectures, 15 oral communications (shorter lectures) and 20 posters shared by ca. 80 participants. The abstracts of lectures and posters, as well as the winners of the best oral communication and poster awards, are presented in this report.

## 1. Acknowledgments

**Figure 1:**
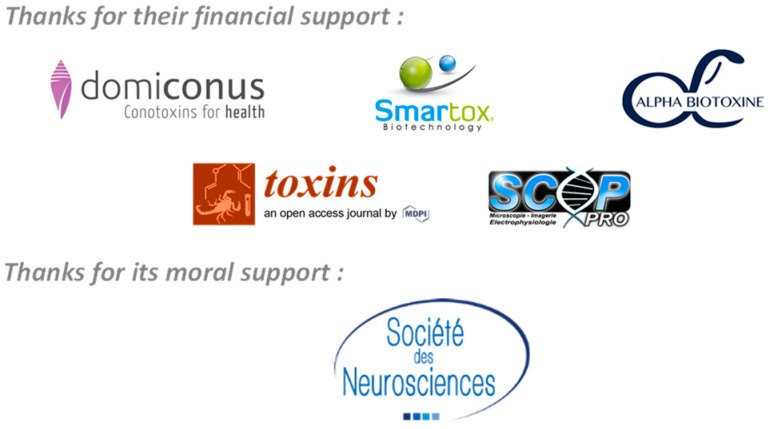
Sponsor logos.

We warmly acknowledge the contribution of all those people who work daily at ensuring the national and international shinning of the French Society of Toxinology (SFET) and those who made the 27th Meeting on Toxinology a success. We also address special thanks to our sponsors who, this year again, supported our meeting (Figure 1).

## 2. Preface

Toxins are biologically active substances produced by most kinds of living organisms, bacteria, fungi, plants and animals. They present a vast diversity of molecular structures and target a wide variety of receptors involved in a range of physiological processes. As toxins were selected during evolution to acquire/improve their disabling/lethal effect, they display finely tuned functional properties often associated with high affinities and selectivity. Moreover, toxins are valuable tools to unravel cellular processes due to their extreme specificity for cell surface and/or intracellular targets. Therefore, toxins are very attractive compounds because of their Janus-like character, being both poisons and remedies, and as such, they have been primarily investigated not only for the light they can throw on fundamental physiological processes but also for their potential therapeutic applications.

This 27th Meeting on Toxinology of the SFET was held on 9–10 December 2021 as a virtual meeting (e-RT27). The central theme selected for this meeting, “Toxins: Mr Hyde or Dr Jekyll?”, gave rise to three thematic sessions: the first one on plant toxins, algal toxins and mycotoxins; the second one on animal toxins; and the third one on bacterial toxins, all sessions were aimed at emphasizing the latest findings on this topic. Apart from this central theme, a “miscellaneous” session was dedicated to recent results obtained in Toxinology. Ten speakers from seven countries (Australia, Brazil, Burkina Faso, France, Germany, the Netherlands and the United States of America) were invited as international experts to present their work, and other researchers and students presented theirs through 15 shorter lectures and 20 posters. Of the ca. 80 participants who registered, 38% were foreigners, a value highlighting the international attractiveness of the SFET meetings.

**Figure 2:**
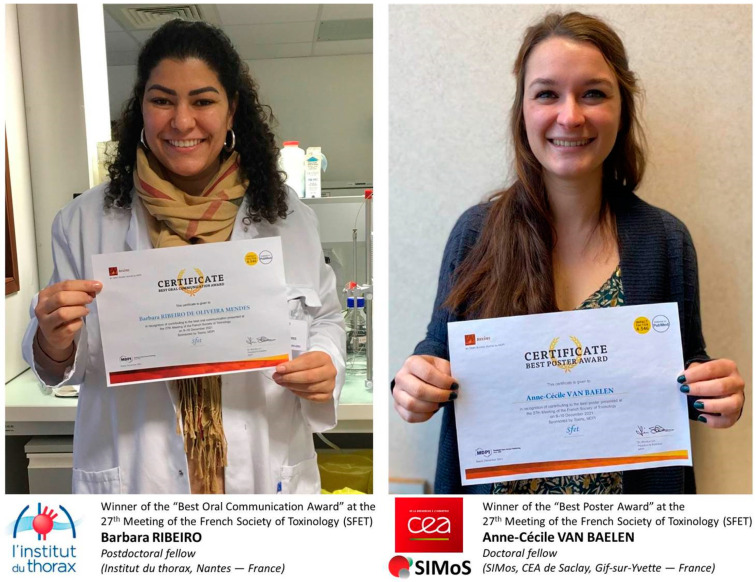
Winners of the “Best Oral Communication” and “Best Poster” Awards at the 27th Meeting of the French Society of Toxinology (SFET).

Owing to a donation from MDPI-Toxins, two prizes of EUR 300 each were awarded to the best oral communication and the best poster (Figure 2), both selected from a vote by all the participants to the meeting.

Last but not least, we warmly thank the Editors of MDPI—Toxins for supporting the publication of a Special Issue also entitled “Toxins: Mr Hyde or Dr Jekyll?”, and gathering this meeting report, along with peer-reviewed original articles and reviews. We believe that this Special Issue will be attractive to all, including those of our colleagues who could not attend the e-RT27 meeting, and that it will represent a comprehensive source of information for researchers and students in Toxinology.

## 3. Scientific and Organizing Committees (SFET Board of Directors)

Julien Barbier, CEA de Saclay, Gif-sur-Yvette, France

Evelyne Benoit, CEA de Saclay, Gif-sur-Yvette, France

Alexandre Chenal, Institut Pasteur, Paris, France

Michel De Waard, L’institut du thorax, Nantes, France

Sylvie Diochot, Institut de Pharmacologie Moléculaire et Cellulaire, Valbonne, France

Sébastien Dutertre, Institut des Biomolécules Max Mousseron, Montpellier, France

Daniel Ladant, Institut Pasteur, Paris, France

Christian Legros, Université d’Angers, Angers, France

Pascale Marchot, CNRS/Aix-Marseille Université, Marseille, France

Gilles Prévost, Institut de Bactériologie, Université de Strasbourg, France

Michel R. Popoff, Institut Pasteur, Paris, France

Loïc Quinton, Université de Liège, Liège, Belgium

## 4. Invited Lectures (When More Than One Author, the Underlined Name Is That of the Presenter)

### 4.1. The Plant with the Scorpion Sting: Novel Pain-Causing Toxins from the Australian Giant Stinging Tree

Irina Vetter ^1,4,^*, Edward K. Gilding ^1^, Sina Jami ^1^, Jennifer R. Deuis ^1^, Mathilde R. Israel ^1,2^, Peta J. Harvey ^1^, Aaron G. Poth ^1^, Fabian B.H. Rehm ^1^, Jennifer L. Stow ^1^, Samuel D. Robinson ^1^, Kuok Yap ^1^, Darren L. Brown ^1^, Brett R. Hamilton ^3^, David Andersson ^2^, David J. Craik ^1^, Thomas Durek ^1^

^1^ Institute for Molecular Bioscience, The University of Queensland, Brisbane, QLD 4072, Australia.

^2^ Wolfson Centre for Age-Related Diseases, Institute of Psychiatry, Psychology & Neuroscience, King’s College London, London, SE5 8AF, United Kingdom.

^3^ Centre for Advanced Imaging, The University of Queensland, Brisbane, QLD 4072, Australia.

^4^ School of Pharmacy, The University of Queensland, Brisbane, QLD 4102, Australia.

* Correspondence: i.vetter@uq.edu.au

**Abstract:** Stinging Trees from Australasia produce remarkably persistent and painful stings upon contact of their stiff epidermal hairs, called trichomes, with mammalian skin. *Dendrocnide*-induced acute pain typically lasts for several hours, and intermittent painful flares can persist for days and weeks. Our recent work shows that the venoms of Australian *Dendrocnide* species contain heretofore-unknown pain-inducing peptides that potently activate sensory neurons and delay the inactivation of voltage-gated sodium channels. These neurotoxins localize specifically to the stinging hairs and are mini-proteins of 4 kDa whose 3D structure is stabilized in an inhibitory cystine knot motif, a characteristic shared with neurotoxins found in spider and cone snail venoms. Our results provide an intriguing example of inter-kingdom convergent evolution of animal and plant venoms with shared modes of delivery, molecular structure and pharmacology.

**Keywords:** pain; peptide toxin; sodium channel; stinging nettle

### 4.2. Evidences That Pinnatoxin-G Crosses Intestinal, Hemato-Encephalic and Maternofetal Barriers to Reach Central and Peripheral Nicotinic Acetylcholine Receptors

Denis Servent ^1,^*, Carole Malgorn ^1^, Sophie Gil ^2^, Christelle Simasotchi ^2^, Anne-Sophie Hérard ^3^, Thierry Delzescaux ^3^, Robert Thai ^1^, Peggy Barbe ^1^, Mathilde Keck ^1^, Fabrice Beau ^1^, Armen Zakarian ^4^, Vincent Dive ^1^, Jordi Molgó ^1^

^1^ Université Paris-Saclay, CEA, Département Médicaments et Technologies pour la Santé (DMTS), Service d’Ingénierie Moléculaire pour la Santé (SIMoS), ERL CNRS 9004, F-91191 Gif-sur-Yvette, France.

^2^ Université de Paris, UMR S1139, Faculté de Pharmacie de Paris, France.

^3^ Université Paris-Saclay, UMR 9199, CNRS, CEA, MIRCen, Fontenay-aux-Roses, France.

^4^ University of California Santa Barbara, Department of Chemistry and Biochemistry, California 93106, USA.

* Correspondence: denis.servent@cea.fr

**Abstract:** The warming of ocean temperatures and the increase in nutrients has driven an intensification of harmful algal bloom events. During these active dinoflagellate proliferations, phycotoxins may accumulate in shellfish tissues and can be transferred into fish, marine mammals and ultimately to humans. Cyclic imines, produced by various species of marine dinoflagellates, constitute a widely distributed group of phycotoxins, including pinnatoxins. Pinnatoxin-G (PnTx-G) produced by the cosmopolitan dinoflagellate *Vulcanodinium rugosum* is considered the precursor of other pinnatoxins, and it is a fast-acting toxin in the mouse bioassay. PnTx-G is regularly detected in European coastal environments and in contaminated shellfish samples and may represent a human health risk. In this work, exploiting the ability of PnTx-G to be chemically synthesized and radiolabeled, we studied in vivo the toxicokinetics of [^3^H]-PnTx-G and its capacity to interact with neuronal and muscle-type nicotinic acetylcholine receptors (nAChRs). The biodistribution of [^3^H]-PnTx-G, using high-resolution digital radio-imaging after oral or intravenous administration to rats, revealed the presence of the radiolabeled toxin in various peripheral organs, as well as in specific regions of the central nervous system, highlighting its property to cross both the intestinal and blood–brain barriers. In addition, we demonstrate that PnTx-G crosses the materno–fetal barrier, first in rats by detecting the radiolabeled toxin in embryos after its injection to pregnant rats and secondly in humans, using a perfused ex vivo cotyledon model and mass spectrometry analysis. Furthermore, to assess the nAChR subtypes labeled by [^3^H]-PnTx-G, we performed competition experiments on brain and embryos sections in the presence of selective nAChR antagonists, revealing the major role of muscle-type and α7 subtype in the peripheral and central labeling, respectively. In conclusion, this work shows that PnTx-G efficiently crosses the intestinal, blood–brain and placental barriers to interact with central and peripheral nAChRs, supporting its in vivo effects. The mechanism used by pinnatoxins to cross these physiological barriers and the possible involvement of a receptor-mediated process are still under investigation.

**Keywords:** biodistribution; nicotinic acetylcholine receptor; pinnatoxin

### 4.3. A Blue Mountains Funnel-Web Spider Toxin Expressed under Control of a Hemolymph-Specific Promoter Increases Fungal Lethality against Insecticide-Resistant Malaria-Vector Mosquitoes

Étienne Bilgo ^1,^*, Brian Lovett ^2^, Raymond St. Leger ^2^, Abdoulaye Diabate ^1^

^1^ Institut de Recherche en Sciences de la Santé/Centre Muraz, Bobo-Dioulasso, Burkina Faso.

^2^ Department of Entomology, University of Maryland, College Park, MD 20742, USA.

* Correspondence: bilgo02@yahoo.fr

**Abstract:** The continued success of malaria control efforts requires the development, study and implementation of new technologies that circumvent insecticide resistance. We previously demonstrated that fungal pathogens could provide an effective delivery system for mosquitocidal or malariacidal biomolecules. In this study, we firstly compared genes from arthropod predators encoding insect-specific sodium, potassium and calcium channel blockers for their ability to improve the efficacy of Metarhizium against wild-caught, insecticide-resistant anophelines. Toxins expressed under the control of a hemolymph-specific promoter increased fungal lethality to mosquitoes at spore dosages as low as one conidium per mosquito. One of the most potent, the EPA-approved Hybrid (Ca^2+^/K^+^ channel blocker), was studied for pre-lethal effects. These included reduced blood-feeding behavior, with almost 100% of insects infected with ca. six spores unable to transmit malaria within five days post-infection, surpassing the World Health Organization threshold for successful vector control agents. Furthermore, recombinant strains co-expressing Hybrid toxin and AaIT (Na^+^ channel blocker) produced synergistic effects, requiring 45% fewer spores to kill half of the mosquitoes in five days as single toxin strains. Secondly, through a semifield trial in a MosquitoSphere (a contained, near-natural environment) in Soumousso, a region of Burkina Faso where malaria is endemic, we confirmed the proof of our concept in the field. The expression of Hybrid toxin increased fungal lethality and the likelihood that insecticide-resistant mosquitoes would be eliminated from this site. In summary, our results identified a repertoire of toxins with different modes of action that improve the utility of entomopathogens as a technology that is compatible with existing insecticide-based control methods.

**Keywords:** biotechnology; malaria; mosquito; mycology; toxin

### 4.4. Bee Venom and Its Component Apamin Have Anti-Parkinsonian Properties in Animal Models of Parkinson’s Disease

Marianne Amalric ^1,^*, Nathalie Turle-Lorenzo ^1^, Christophe Melon ^2^, Nicolas Maurice ^2^

^1^ Laboratoire de Neurosciences Cognitives (LNC) UMR 7291, CNRS, Aix-Marseille Univ., Marseille, France.

^2^ Institut de Biologie du Développement de Marseille (IBDM) UMR 7288, NeuroMarseille, Aix-Marseille Univ., CNRS, Marseille, France.

* Correspondence: marianne.amalric@univ-amu.fr

**Abstract:** Parkinson’s disease (PD) is an age-related neurological disorder that affects more than 6.3 million people worldwide. PD is characterized by progressive loss of dopaminergic (DA) neurons in the substantia nigra (SN), which leads to a wide range of debilitating motor, cognitive and neuropsychiatric symptoms. L-DOPA therapy is effective on motor symptoms and motor fluctuations but leads over the years to dyskinesia. Calcium-activated potassium channels (SK channels) have recently emerged as alternative therapeutic targets because they regulate the neuronal firing of midbrain dopamine neurons. SK channels (in particular SK_2_/SK_3_ subtypes) are highly expressed in the basal ganglia (BG). These brain structures are dysregulated by DA neuronal degeneration. Potassium channels are privileged targets because they regulate neuronal excitability in order to maintain balance in the input-output circuits of the BG. Our study aimed to assess the behavioral effects of apamin, a selective SK_2_/SK_3_ channel blocker peptide extracted from bee venom, and compare them with those produced by bee venom, in pharmacological and 6-hydroxydopamine (6-OHDA) lesional models of PD in rats. Both bee venom and apamin reversed haloperidol-induced catalepsy, a model of akinetic symptoms, while a co-treatment with the SK opener CYPPA prevented this antiakinetic effect. After extensive unilateral 6-OHDA nigrostriatal lesions, acute and subchronic administration of bee venom and apamin reduced forelimb asymmetry in the cylinder test and apomorphine-induced rotations revealing an antiparkinsonian action on motor symptoms. In another rat model of partial bilateral DA nigrostriatal lesions, apamin also reduced deficits revealed on anxiety, social interaction and visuospatial memory. The neural substrates of these effects were investigated by in vivo electrophysiological recordings of neuronal activity of the BG output structure substantia nigra pars reticulata (SNr). Bee venom restored the balance between the inhibitory and excitatory influence exerted by the trans-striatal direct and indirect pathways that were disrupted by the pharmacological blockade of DA receptors. These results suggest that bee venom and apamin restore the functional properties of the basal ganglia circuitry in PD conditions and emphasize the crucial role of potassium channels (Ca^2^-dependent) in these anti-parkinsonian effects. Supported by CNRS, AMU, Fondation de France, Association France Parkinson.

**Keywords:** bee venom; dopamine; Parkinson’s disease

### 4.5. Slithering Stem Cells—Understanding Snake Venom Production Using Organoids

Jens Puschhof ^1,2,^*, Yorick Post ^1^, Joep Beumer ^1^, Harald M. Kerkkamp ^3^, Julien Slagboom ^4^, Buys De Barbanson ^1,2^, Nienke R. Wevers ^5^, Xandor Spijkers ^5,6^, Thomas Olivier ^5^, Taline D. Kazandijan ^7^, Stuart Ainsworth ^7^, Carmen Lopez Iglesias ^8^, Willine van de Wetering ^1,8^, Maria C. Heinz ^2,9^, Ravian L. Van Ineveld ^2,10^, Regina G.D.M. Van Kleef ^11^, Harry Begthel ^1^, Jeroen Korving ^1^, Yotam E. Bar-Ephraim ^1,2^, Walter Getreuer ^12^, Anne C. Rios ^2,10^, Remco H.S. Westerink ^11^, Hugo J.G. Snippert ^2,9^, Alexander Van Oudenaarden ^1,2^, Peter J. Peters ^8^, Freek J. Vonk ^3^, Jeroen Kool ^4^, Michael K. Richardson ^3^, Nicholas R. Casewell ^7^, Hans Clevers ^1,2,10^

^1^ Hubrecht Institute, Royal Netherlands Academy of Arts and Sciences (KNAW) and UMC Utrecht, 3584 CT Utrecht, The Netherlands.

^2^ Oncode Institute, Hubrecht Institute, 3584 CT Utrecht, The Netherlands.

^3^ Institute of Biology Leiden, Department of Animal Science and Health, 2333 BE Leiden, The Netherlands.

^4^ Division of BioAnalytical Chemistry, Department of Chemistry and Pharmaceutical Sciences, Vrije Universiteit Amsterdam, 1081 LA Amsterdam, The Netherlands.

^5^ Mimetas BV, Organ-on-a-chip Company, 2333 CH Leiden, The Netherlands.

^6^ Department of Translational Neuroscience, University Medical Center, 3584 CG, Utrecht, The Netherlands.

^7^ Centre for Snakebite Research & Interventions, Parasitology Department, Liverpool School of Tropical Medicine, Liverpool, L3 5QA, UK.

^8^ The Maastricht Multimodal Molecular Imaging institute, Maastricht University, 6229 ER Maastricht, The Netherlands.

^9^ Molecular Cancer Research, Center for Molecular Medicine, University Medical Center Utrecht, Utrecht University, 3584 CX Utrecht, The Netherlands.

^10^ The Princess Maxima Center for Pediatric Oncology, 3584 CS Utrecht, The Netherlands.

^11^ Neurotoxicology Research Group, Division of Toxicology, Institute for Risk Assessment Sciences (IRAS), Utrecht University, 3584 CL Utrecht, The Netherlands.

^12^ Serpo, 2288 ED Rijswijk, The Netherlands.

* Correspondence: jens.puschhof@dkfz-heidelberg.de

**Abstract:** Recent advances in organoid technology have proven this system to be a valuable tool in understanding human organ development and pathologies. These adult stem cell-derived cultures closely recapitulate the structural and functional properties of their organ of origin. Here, we expand the organoid technology toolbox by describing a protocol to culture non-mammalian organoids derived from a snake venom gland. The complexity of venom production, composition and function remain largely unknown for many species. Organoids derived from an *Aspidelaps lubricus* venom gland can be long-term expanded and histologically resemble the gland. Expression of typical venom-related transcripts (three-finger toxins and Kunitz-type protease inhibitors) can be detected in proliferating organoids with RNA sequencing. Single-cell RNA sequencing reveals distinct venom-expressing cell types, as well as proliferating cells with features of mammalian stem cells. By using mass spectrometry, we identified peptides in the culture medium supernatant that match the composition of the crude venom of the same species. Venom gland organoids furthermore consist of specialized secretory cells visible by transmission electron microscopy. The system enables the investigation of venom production and function on a cellular level in controlled conditions and without the need for experimental animals. This study describes the adaption of organoid technology to a non-mammalian species, providing a model to understand the complexity of the snake venom gland.

**Keywords:** in vitro; organoid; snake; venom gland

### 4.6. Prospection of Animal Toxins in Drug Discovery—Challenges and Perspectives

Karla de Castro Figueiredo Bordon *

University of São Paulo, School of Pharmaceutical Sciences of Ribeirão Preto, Ribeirão Preto, São Paulo, Brazil.

* Correspondence: karlabordon@yahoo.com.br

**Abstract:** Venomous animals may cause severe medical complications and untimely death, but their venoms are also sources of molecules acting on the nervous, cardiovascular, immune and other systems. Animal toxins are often used as pharmacological tools for the validation of therapeutic targets, but they are also used as cosmeceuticals and diagnostic tools in the design of new therapeutic agents and to improve drug libraries. Some drugs used in the therapy of many disorders, such as diabetes and cardiovascular diseases, were developed based on the molecular structures of animal toxins. Captopril was the first animal toxin-based drug approved for human use. Scorpion neurotoxins are known to be responsible for the pathological manifestations of scorpionism. They are a threat to human health but may serve as leads for the development of new therapies against current and emerging diseases. The scorpion toxin chlorotoxin is undergoing clinical phase trials as a fluorescent molecular probe that paints tumors, while the CPP-Ts peptide is a potential intranuclear delivery tool targeting cancer cells. The bioprospection of neurotoxins that block KV1.3 potassium channels may lead to the development of drugs to treat autoimmune diseases since these channels are found in macrophages, platelets and T cells. The overexpression of KV1.3 in lymphocytes is related to diseases such as atherosclerosis, some types of cancers, obesity and autoimmune diseases such as Crohn’s disease, dermatitis, psoriasis and rheumatoid arthritis, among others. Related to snake toxins, a heterologous fibrin sealant, comprising a cryoprecipitate rich in fibrinogen extracted from the blood of buffaloes in association with a serine protease from rattlesnake, is a biodegradable biological product that reduces surgical time, promotes faster postoperative recovery, is highly adhesive and can also be used as an adjuvant in suture procedures and as a carrier for drug delivery. The use of biotechnological tools, such as the heterologous expression of peptides and proteins, enables the production of biologically active molecules in sufficient quantity for the evaluation and development of new medicines. Recently, a serine protease was recombinantly expressed with functional and structural integrity and showed fibrinogenolytic activity and inhibition against the hEAG1 channel, highly expressed in tumor cells, in a mechanism independent of its catalytic activity. Non-PEGylated and PEGylated forms of this enzyme share similar kinetic and functional characteristics, and the latter showed no evidence of immunogenicity or cytotoxicity, even at high concentrations. Given the examples, approaches to improve the druggability of animal toxins are fruitful fields for future research.

**Keywords:** biological dressing; heterologous expression; immunosuppression; PEGylation; poison; toxin

### 4.7. Redirecting the Target of Muscarinic Toxin

Shoji Maeda ^1,7,^*, Jun Xu ^1^, Francois Marie N. Kadji ^2^, Mary J. Clark ^3^, Jiawei Zhao ^4^, Naotaka Tsutsumi ^5^, Junken Aoki ^2^, Roger K. Sunahara ^3^, Asuka Inoue ^2^, K. Christopher Garcia ^1,5,6^, Brian K. Kobilka ^1^

^1^ Stanford University School of Medicine, Stanford, CA 94305, USA;

^2^ Tohoku University, Sendai, Japan;

^3^ University of California San Diego School of Medicine, La Jolla, CA 92093, USA;

^4^ Tsinghua University, Beijing, China;

^5^ Stanford University School of Medicine, Stanford, CA 94305, USA;

^6^ Howard Hughes Medical Institute; ^7^ University of Michigan, MI 48109, USA.

* Correspondence: shojim@umich.edu

**Abstract:** Muscarinic acetylcholine receptors are prototypical class-A GPCRs that are distributed throughout the human body and play critical roles in the maintenance of fundamental human physiology by responding to the neurotransmitter acetylcholine. Five muscarinic receptor subtypes (M1R–M5R) have been identified in humans with distinct G protein coupling preferences, distribution profile and physiological roles. With their conserved orthosteric ligand-binding site and the presence of an allosteric ligand-binding site at the extracellular region, muscarinic receptors were extensively studied as a model system for the subtype-selective targeting at a GPCR through small molecule allosteric modulators. Animals have evolved venoms to hunt prey and/or run from predators. These venoms have been a rich source to isolate natural products that modulate numerous protein functions, including GPCRs. Peptide and small protein toxins are emerging modalities because of their superior selectivity and stability. Muscarinic toxin belongs to the three-finger toxin family and is one of the best-characterized peptide toxins produced by venomous snakes. A member of muscarinic toxin, MT7, shows extremely high selectivity to M1R over other muscarinic receptors and elicits an inhibitory effect. We solved the crystal structure of the M1R–MT7 complex and revealed the exquisite design of this toxin to exclusively fit into M1R by selectively engaging at the allosteric site with residues unique to M1R. By using the structural information and the surface display platform, we succeeded in converting the subtype selectivity of MT7 to another muscarinic receptor, M2R. Furthermore, we obtained conformational selective three-finger proteins exclusively targeting the active conformation of muscarinic receptors. Our data indicate a promise of repurposing this natural toxin scaffold to a broader range of target systems.

**Keywords:** muscarinic acetylcholine receptor; protein engineering; three-finger toxin

### 4.8. Synthetic AB-Toxins for Targeted Pharmacological Modulation of Cancer and Immune Cells

Holger Barth *

University of Ulm Medical Center, Institute of Pharmacology and Toxicology, Albert-Einstein-Allee 11, 89081 Ulm, Germany.

* Correspondence: holger.barth@uni-ulm.de

**Abstract:** Bacterial AB-toxins are highly toxic proteins because of their unique modular structure: these toxins bind to mammalian cells by a specific receptor binding (B) subunit and deliver their enzymatically active (A) subunit into the host cell cytosol via their intrinsic translocation (T) subunit. In the cytosol, the A subunit modifies its specific cellular substrate, which alters cellular structures and/or functions and is the reason for the clinical symptoms of the particular toxin-associated disease. Because of their intracellular, highly specific and extremely potent mode of action, some AB-toxins serve as valuable molecular tools in pharmacology and cell biology for the targeted modulation of cell functions and are used as drugs. In past years, we and others used the non-toxic B and T subunits of various toxins to deliver pharmacologically active proteins (e.g., enzymes) and peptides into the cytosol of cells and developed cell type-selective transport systems to modulate functions of immune and cancer cells. However, for some cell types, there are no selective AB toxins available by nature, and therefore, we aimed to develop “synthetic” AB-toxins. In one approach, supramolecular transporter systems were generated, where avidin with four biotin-binding sites serves as a central binding platform for three biotinylated binding peptides that selectively bind to receptors on target cells and trigger internalization of the transporter molecule, and biotinylated cargo molecules that act as “A subunit” in the cytosol. Proof-of-concept was provided by using clostridial C3 Rho-inhibitor as cargo enzyme and the peptide somatostatin as a ligand to selectively target human lung cancer cells in vitro and in the HET/CAM model for a human xenograft lung tumor that overexpresses somatostatin receptor. This strategy also served for the generation of a novel cell type-selective Rho-inhibitor for human neutrophils. This universal “LEGO-like” modular approach represents a promising platform for the generation of cell-type selective synthetic AB-toxins for the targeted pharmacological modulation of cell functions.

**Keywords:** bacterial AB-toxin; cellular uptake; targeted drug delivery; receptor binding; membrane translocation; avidin-biotin technology; (semi)synthetic AB-type molecule

### 4.9. Phenotypic Screening for Anti-Toxin Molecules Selects Inhibitors of Intracellular Trafficking with Broad-Spectrum Anti-Infectious Properties

Daniel Gillet *

Université Paris-Saclay, CEA, INRAE, Département Médicaments et Technologies pour la Santé (DMTS), SIMoS, 91191 Gif-sur-Yvette, France.

* Correspondence: daniel.gillet@cea.fr

**Abstract:** In the aftermath of the anthrax letter attacks in the fall of 2001, the French authorities mandated the French health products sanitary safety agency (AFSSAPS) to determine the needs for medical countermeasures (MCM) against biothreat agents. A working group under the lead of Prof. Dominique Dormont established the cruel lack of medications against bacterial and plant toxins prone to be used as bioweapons, among which the plant toxin ricin. Then, the growing concerns about antimicrobial resistance led to suggest that antitoxin drugs should be developed to treat common public health bacterial infections as an alternative to antibiotics. In these respects, we developed two phenotypic screenings of small chemical molecules in 2005 and 2015; the first was against ricin toxin, and the second (in collaboration with Emmanuel Lemichez, Institut Pasteur) was against the cytotoxic necrotizing factor CNF1, produced by uropathogenic and extra-intestinal strains of *Escherichia coli*. The first screening was designed to select molecules capable of rescuing cells from ricin-induced toxicity. The second screening was designed to select molecules inhibiting the degradation of CNF1-induced activated Rac1. Overall, the anti-ricin screening identified three original inhibitors of intracellular trafficking. Retro-1 and Retro-2 act on the retrograde trafficking pathway (collaboration with Ludger Johannes, Institut Curie). In particular, Retro-2 interferes with the ERES protein Sec16A and the circulation of syntaxin-5 along the anterograde and retrograde routes. ABMA acts on the endolysosomal pathway by the accumulation of late endosomes and alteration of the autophagic flux. The CNF1 screening identified C910 as an original inhibitor of early endosome sorting function. Most interestingly, all four inhibitors displayed a broad spectrum of inhibition against a series of bacterial toxins, but also viruses, bacteria and parasites exploiting these pathways for intoxication or infection of cells. There are at least eight examples published or under the submission of in vivo protection of mice by these compounds against intoxications and/or infections. Compound optimization by medicinal chemistry was performed. The challenges of turning these molecules into drugs are discussed. Nevertheless, 16 years of research by us and others showed that intracellular trafficking pathways might be interesting druggable targets to fight intracellular toxins and pathogens.

**Keywords:** bacterial toxin; broad-spectrum; intracellular trafficking; ricin toxin; small chemical inhibitor

### 4.10. NLRP3 Inflammasome Sensing of RhoGTPase-Activating Toxins during Bacteremia

Laurent Boyer *

C3M, INSERM U1065, Université Cote d’Azur, Nice, France.

* Correspondence: boyerl@unice.fr

**Abstract:** The detection of the activities of pathogen-encoded virulence factors by the innate immune system has emerged as a new paradigm of pathogen recognition. Much remains to be determined regarding the molecular components contributing to this defense mechanism in mammals and its importance during infection. Our team showed the central role of the IL-1β signaling axis in controlling the Escherichia coli burden in the blood in response to the sensing of the RhoGTPase-activating toxin CNF1. Using the CNF1 toxin, we provided evidence of the role of the NLRP3 inflammasome in sensing the activity of bacterial toxins and virulence factors that activates host RhoGTPases. We demonstrated that this activation relies on monitoring of the toxin activity on the RhoGTPase Rac2. We also showed that the NLRP3 inflammasome is activated by a signaling cascade involving the P21-activated kinases (Pak)-1/2. The Pak1-mediated phosphorylation of threonine-659 of NLRP3 was necessary for NLRP3-Nek7 interaction, inflammasome activation and IL-1ß cytokine maturation. Furthermore, inhibition of the Pak1-NLRP3 axis diminished the bacterial clearance of CNF1-expressing E. coli during bacteremia. Altogether, our results established Pak1/2 as critical regulators of the NLRP3 inflammasome and revealed the role of the Pak1-NLRP3 signaling axis in vivo during bacteremia.

**Keywords:** bacterial toxin; inflammasome; NLPR3 RhoGTPase

## 5. Oral Presentations (When More Than One Author, the Underlined Name Is That of the Presenter)

### 5.1. Screening an In-House Library of Isoquinoline Alkaloids with Fluorescent Probes for Discovering New Ligands of Voltage-Gated Na^+^ and Ca^2+^ Channels

Claire Legendre ^1,^*, Jacinthe Frangieh ^1,2^, Léa R **é** **thor** **é** **^1^** **, Quentin Coquerel ^1^, Daniel Henrion ^1^, César Mattei ^1^, Ziad Fajloun ^2,4^, Anne-Marie Le Ray ^3^, Christian Legros ^1^**

^1^ Laboratory Mitochondrial and Cardiovascular Pathophysiology (MITOVASC), CNRS UMR 6015—INSERM U1083, CHU Angers, Université d’Angers, Angers, France.

^2^ Laboratory of Applied Biotechnology (LBA3B), Azm Center for Research in Biotechnology and its Applications, EDST, Lebanese University, 1300 Tripoli, Lebanon.

^3^ SONAS Laboratory, SFR QUASAV, Université d’Angers, Angers, France.

^4^ Department of Biology, American University of Beirut, 11-0236 Beirut, Lebanon.

* Correspondence: claire.legendre@univ-angers.fr

**Abstract:** The isoquinoline alkaloids (IA) is a large chemical group of natural compounds with various structures and acting on multiple pharmacological targets of therapeutical interest, including ion channels, such as voltage-gated Na+ (Nav) and Ca2+ (Cav) channels. These ion channels are established molecular targets for the development of therapeutical agents in cardiovascular diseases and particularly arrhythmia. Here, we screened 62 IA from an in-house vegetal chemical library, using a model of excitable cells, the rat pituitary GH3b6 cells, which endogenously express both Nav and Cav channels, to find new blockers for these channels. Moreover, we demonstrated that Nav activation by selective neurotoxins induced an intracellular Ca^2+^ concentration [Ca^2+^]_i_ elevation mediated by Cav channels, highlighting crosstalk between Nav and Cav channels that we used for pharmacological studies. We first tested these IA for their abilities to inhibit batrachotoxin (BTX)-induced depolarization using fluorescent voltage-sensor probes (VSP) and identified two oxoaporphines, namely liriodenine and oxostephanine, which abolished BTX-induced VSP signal with IC_50_ values in the micromolar range. While the blocking activity of liriodenine was already reported, the activity of oxostephanine on Nav channels has never been described yet. Interestingly, oxostephanine differs from liriodenine only by a methoxy group on the benzyl part of the skeleton. This subclass of IA might constitute a new group of ligands of Nav channels. We confirmed the blocking effect of both molecules in a Na+ influx assay using the Na^+^ fluorescent probe ANG-2. Since liriodenine is also known to block Cav channels, we hypothesized that oxostephanine probably targets Cav channels. Thus, we investigated the effects of both IA on L-type Cav channels (LTCC) expressed in GH3b6 cells. In order to activate LTCC, we used a chemical depolarization with KCl or the agonist Bay K8644 and monitored [Ca^2+^]_i_ change with the fura-2 probe. Our results showed that liriodenine and oxostephanine induced a concentration-dependent inhibition of KCl- and Bay K8644-evoked Ca^2+^ responses, with similar IC_50_ values in the micromolar range. In addition, this interaction of liriodenine and oxostephanine on LTCC was also highlighted by their ability to inhibit veratridine (VTD)- and BTX-induced [Ca^2+^]_i_ elevation. In conclusion, our data showed that liriodenine and oxostephanine, two oxoaporphine alkaloids, inhibit Nav and Cav channels with similar potency. The oxoaporphine skeleton might bring an interesting pharmacophore for structure-function relationship studies for designing more selective ligands toward Cav and Nav channels and for developing new antiarrhythmic therapeutical leads.

**Keywords:** Cav channel; isoquinoline alkaloid; Nav channel

### 5.2. The Venom of the Lebanese Viper, Montivipera bornmuelleri, Contains Vasoactive Compounds

Jacinthe Frangieh ^1,2,^*, Joohee Park ^1^, Ziad Fajloun ^2^, Loïc Quinton ^3^, Riyad Sadek ^4^, Daniel Henrion ^1^, César Mattei ^1^, Christian Legros ^1^

^1^ Laboratory Mitochondrial and Cardiovascular Pathophysiology—MITOVASC, CNRS UMR 6015, INSERM U1083, CHU Angers, Université d’Angers, Angers, France.

^2^ Laboratory of Applied Biotechnology (LBA3B), Azm Center for Research in Biotechnology and its Applications, EDST, Lebanese University, 1300 Tripoli, Lebanon.

^3^ Laboratory of Mass Spectrometry, Department of Chemistry, MolSys Research Unit, University of Liège, 4000 Liège, Belgium.

^4^ Department of Biology, American University of Beirut, 11-0236 Beirut, Lebanon.

* Correspondence: jacynthefrangieh@gmail.com

**Abstract:** Snake venoms are rich mixtures of polypeptides that target, among other physiological networks, the cardiovascular system. Snake toxins such as phospholipase A2, natriuretic peptides, and bradykinin-potentiating peptides exert various effects on the cardiovascular system (hypotension, vasorelaxation, etc.). Some of these toxins were developed as drugs with antihypertensive properties, such as Captopril™. We previously showed that the venom of the viper *Montivipera bornmuelleri* induces relaxation of rat aortic rings. Here, we aim to identify and characterize the vasoactive compounds of this venom. We fractionated the venom by HPLC. The fractions were assayed on the endothelial MS1 cell line and CHO cells lines expressing muscarinic receptors M1, M3 or M5. We screened for compounds able to inhibit acetylcholine-induced intracellular Ca^2+^ rise, using the fluorescence probe FURA-2. In addition, wire myography on isolated mesenteric arteries was used. Proteomic analysis was performed to characterize selected fractions. The crude venom exhibited a vasorelaxant effect on mesenteric arteries. Among 23 fractions of *Mb* venom, one fraction, namely P14, was selected for its ability to reduce acetylcholine-induced Ca^2+^ rise in MS1 cells. The proteomic analysis allowed us to identify a novel peptidyl toxin (P14) as a homolog of the vascular endothelial growth factor (VEGF). However, P14 was unable to antagonize acetylcholine-induced Ca^2+^ rise in cells expressing muscarinic receptors, suggesting that this peptide interferes with other target(s) of the Ca^2+^ signaling pathway in endothelial cells. Further studies will be carried out to characterize the molecular targets of P14 and its effects on vascular function.

**Keywords:** calcium signaling; *Montivipera bornmuelleri* venom; vascular function

### 5.3. Lipids and Cholesterol Mediate the Cytotoxicity of Spider Peptides

Javier Moral-Sanz ^1^, Sergey Kurdyukov ^2^, Ana Vela-Sebastián ^1^, Zoltan Dekan ^3^, Thomas Kremsmayr ^4^, Markus Muttenthaler ^3,4^, Paul F. Alewood ^3^, Gregory G. Neely ^2^, Évelyne Deplazes ^3^, Maria P. Ikonomopoulou ^1,3,^*

^1^ Madrid Institute for Advanced Studies in Food, Madrid, E28049, Spain.

^2^ University of Sydney, Camperdown, NSW 2006, Australia.

^3^ The University of Queensland, St. Lucia, QLD 4072, Australia.

^4^ University of Vienna, Vienna 1090, Austria.

* Correspondence: maria.ikonomopoulou@imdea.org

**Abstract:** Spider gomesin peptides target melanoma cells of BRAF-mutation with minimum effect on non-transformed neonatal foreskin fibroblasts. We hypothesized that the selectivity of gomesin peptides towards melanoma of BRAF mutation could be influenced by interactions with lipids or cholesterol present in cell membranes. In order to elucidate upon this, we employed a multidisciplinary approach, including CRISPR/Cas9 genome-wide screening, to identify key players underlying the antiproliferative mechanisms of gomesins. We also used a combination of fluorescence spectroscopy to measure membrane dipole changes, electrical impedance spectroscopy (EIS) with tethered bilayer membranes (tBLMs) to quantify alterations in membrane conductance and membrane thickness, and cell viability assays. Gomesin and variants showed weak binding to POPC membranes alone. However, the presence of POPS and cholesterol significantly improved the binding of the peptides and lessened membrane disruption. In addition, the cytotoxicity of gomesin was blunted by increasing concentrations of cholesterol in both melanoma cells and fibroblasts. Conversely, cholesterol depletion potentiated the cytotoxicity of peptides in fibroblasts to almost the levels originally observed in melanoma cells. In conclusion, we postulate a specific role of cholesterol in the selective cytotoxic profile of gomesin in melanoma of BRAF mutation.

**Keywords:** cytotoxicity; melanoma; spider peptide

### 5.4. ExoU and ExoY, Two Toxins from Pseudomonas aeruginosa with Different Mechanisms of Action and Biological Effects

Vincent Deruelle ^1,^*, Dorothée Raoux-Barbot ^1^, Undine Mechold ^1^, Philippe Huber ^2^

^1^ Unité de Biochimie des Interactions Macromoléculaires, Institut Pasteur, Département de Biologie Structurale et Chimie, CNRS UMR 3528, Paris, France.

^2^ Center for Immunology of Viral, Auto-immune, Hematological and Bacterial diseases (IMVA-HB/IDMIT), Université Paris-Saclay, INSERM, CEA, Fontenay-aux-Roses, France.

* Correspondence: vincent.deruelle@pasteur.fr

**Abstract:***Pseudomonas aeruginosa* is a ubiquitous and opportunistic Gram-negative bacterium. It is a leading cause of nosocomial infections and is responsible for acute and chronic infections thanks to a broad panel of virulence factors. Among them, the Type 3 Secretion System (T3SS) plays an essential role in the pathogenicity of the bacterium. This system pierces the cell membrane and allows the delivery of toxins into the host cytoplasm. Four T3SS effectors have been identified—ExoS, ExoU, ExoT and ExoY. Most strains inject two or three of these effectors simultaneously, with ExoU and ExoS being mutually exclusive. Each T3SS effector is inactive when injected and requires a host cell factor to be activated. Strains injecting ExoU toxin induce the most severe pathologies because the damage caused by the toxin is rapid and irreversible, leaving no time for proper clinical management. Its mechanism of action is well described. Inside host cells, ExoU is a phospholipase that interacts with a specific lipid at the cytosolic side of the plasma membrane, allowing its activation and leading to cell necrosis. However, several aspects of ExoU activation and its trafficking in human cells upon injection remain elusive. Thanks to genetic approaches and analyses by microscopy, we identified a favored anterograde transport, led by the co-chaperone DNAJC5, which targets ExoU to the plasma membrane. Inactivation of DNAJC5 gene disrupts ExoU-dependent toxicity in vitro and in vivo. Unlike ExoU, the role of ExoY in the pathogenicity of *P. aeruginosa* is still controversial. This toxin is a nucleotidyl cyclase that interacts with the eukaryotic filamentous actin (F-actin) to be activated and thereby to massively convert nucleotide triphosphates into their cyclic form. It was reported that ExoS toxicity to the epithelial cells is enhanced in the absence of ExoY and that in many ExoU+ strains, ExoY is not active. Thus, ExoY seems to limit the action of other toxins. We will discuss our advances in exploring the role of ExoY by studying its effects in infected cells.

**Keywords:** CRISPR-Cas9; DNAJC5; ExoU; ExoY; virulence

### 5.5. Moving Forward Tetanus Therapy: Two Exceptionally Potent humAbs Are Effective for Prophylaxis and Treatment in Mice

Marco Pirazzini ^1,^*, Alessandro Grinzato ^1^, Davide Corti ^2^, Sonia Barbieri ^2^, Oneda Leka ^1^, Francesca Vallese ^1^, Marika Tonellato ^1^, Chiara Silacci-Fregni ^3^, Luca Piccoli ^3^, Eaazhisai Kandiah ^4^, Giampietro Schiavo ^5,6^, Giuseppe Zanotti ^1^, Antonio Lanzavecchia ^3,7^, Cesare Montecucco ^1,8^

^1^ Department of Biomedical Sciences, University of Padova, Via Ugo Bassi 58/B, Padova, 35131, Italy.

^2^ Humabs BioMed SA, 6500 Bellinzona, Switzerland.

^3^ Institute for Research in Biomedicine, Università della Svizzera Italiana, 6500 Bellinzona, Switzerland.

^4^ European Synchrotron Radiation Facility, 71 avenue des Martyrs, F-38000 Grenoble, France.

^5^ Department of Neuromuscular Diseases, Queen Square Institute of Neurology, University College London, London, WC1N 3BG, UK.

^6^ UK Dementia Research Institute, University College London, London, WC1E 6BT UK.

^7^ Fondazione Istituto Nazionale Genetica Molecolare c/o Fondazione IRCCS Cà Granda Ospedale Maggiore Policlinico di Milano, Via Francesco Sforza 35, 20122 Milano, Italy.

^8^ Institute of Neuroscience, National Research Council, Via Ugo Bassi 58/B, Padova, 35131, Italy.

* Correspondence: marco.pirazzini@unipd.it

**Abstract:** Tetanus neurotoxin (TeNT) is the causative agent of tetanus, a life-threatening disease of vertebrates, including humans, characterized by neurogenic muscle rigidity and spasticity. Although tetanus can be prevented by a very effective vaccine, a worldwide clinical practice in the emergency rooms is the administration of anti-TeNT immunoglobulins (TIG), which are used both for prophylaxis, to avoid tetanus development in wounded patients, and for therapy, to treat patients already carrying tetanus symptoms. TIG is produced from the blood of hyperimmune individuals, either humans or horses (in developing countries). As such, it exposes patients to several possible side effects, including infections by still-unknown pathogens as well as dangerous anaphylactic reactions. Human monoclonal antibodies (humAbs), which are emerging as superior therapeutics against several diseases, could overcome the drawbacks of TIG. Here, we screened the immortalized memory B cells pooled from the blood of immunized human donors and isolated two humAbs, dubbed TT104 and TT110, which display an unprecedented neutralization ability against TeNT. We determined the epitopes recognized by TT104 and TT110 via cryo-EM and defined how they interfere with the mechanism of neuron intoxication. These analyses pinpointed two novel mechanistic aspects of TeNT activity in neurons and unraveled at the same time the molecular bases of TT104 and TT110 exceptional neutralization ability. Crucially, the combination of TT104 and TT110 display a prophylactic activity in mice when injected long before TeNT, and the two Fab derivatives (TT104-Fab and TT110-Fab) neutralize TeNT in post-exposure experiments. Of note, in both these two paradigms of experimental tetanus, the humAbs and the Fabs show an activity fully comparable to TIG. Therefore, TT104 and TT110 humAbs and their Fab derivatives meet all requirements for being considered for prophylaxis and therapy of human tetanus and are ready for clinical trials.

**Keywords:** monoclonal antibody; spastic paralysis; tetanus neurotoxin; tetanus prophylaxis

### 5.6. CyaA Toxin Cell Invasion Involves Membrane-Active and Calmodulin-Binding Properties of Its Translocation Domain

Alexis Voegele ^1^, Mirko Sadi ^1^, Darragh P. O’Brien ^1^, Pauline Gehan ^2^, Dorothée Raoux-Barbot ^1^, Maryline Davi ^1^, Sylviane Hoos ^1^, Sébastien Brulé ^1^, Bertrand Raynal ^1^, Patrick Weber ^1^, Ariel Mechaly ^1^, Ahmed Haouz ^1^, Nicolas Rodriguez ^2^, Patrice Vachette ^3^, Dominique Durand ^3^, Sébastien Brier ^1^, Daniel Ladant ^1^, Alexandre Chenal ^1,^*

^1^ Institut Pasteur, CNRS UMR 3528, 75015 Paris, France.

^2^ Sorbonne Université, École Normale Supérieure, PSL University, CNRS, Laboratoire des biomolécules, LBM, 75005 Paris, France.

^3^ Université Paris-Saclay, CEA, CNRS, Institute for Integrative Biology of the Cell (I2BC), 91198, Gif-sur-Yvette, France.

* Correspondence: chenal@pasteur.fr

**Figure 3:**
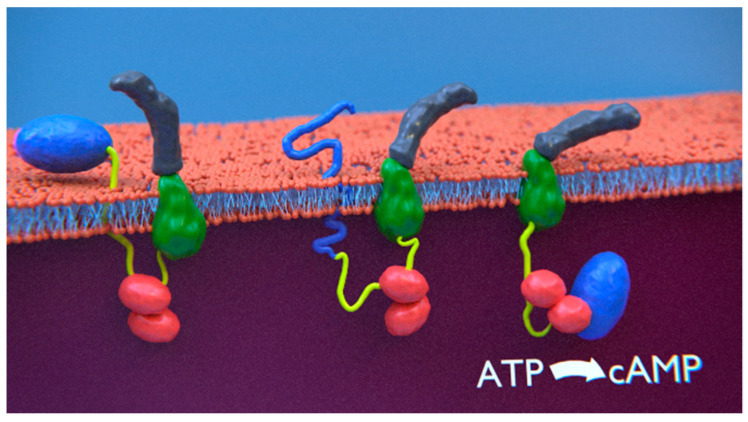
The membrane-active segment (yellow) of the CyaA toxin translocates across the plasma membrane and binds calmodulin (red), which assists the entry and refolding of the catalytic domain (blue) into host cells while the hydrophobic and acylation domains (green) interact with the membrane, and the C-terminal Repeat-in-Toxin domain (grey) remains in the extra-cellular milieu. The cAMP production ultimately leads to cell death.

**Abstract:** The molecular mechanisms and forces involved in the translocation of bacterial toxins into host cells are still a matter of intense research. *Bordetella pertussis*, the causative agent of whooping cough, produces an adenylate cyclase (CyaA) toxin that plays an essential role in the early stages of respiratory tract colonization. CyaA displays a unique intoxication pathway of human cells via a direct translocation of its catalytic domain (AC) across the plasma membrane. Once in the cytosol, AC impairs the physiology of immune cells, leading to cell death. We showed that the P454 peptide (CyaA residues 454–484) is able to translocate across membranes and interact with calmodulin. The key residues involved in membrane-active and calmodulin-binding properties were identified. The mutational analysis demonstrates that these residues play a crucial role in CyaA translocation into target cells. We propose that after CyaA binding to target cells, the P454 segment destabilizes the plasma membrane, translocates across the lipid bilayer and binds calmodulin (Figure 3). Trapping of CyaA by the calmodulin:P454 interaction in the cytosol may assist the entry of AC by converting the stochastic motion of the polypeptide chain through the membrane into an efficient vectorial chain translocation into host cells.

**Keywords:** CyaA; adenylate cyclase toxin; membrane-active peptide; membrane translocation; entropic pulling; calmodulin-binding peptide

**References**

1. Voegele, A., Sadi, M., O’Brien, D. P., Gehan, P., Raoux-Barbot, D., Davi, M., Hoos, S., Brûlé, S., Raynal, B., Weber, P., Mechaly, A., et.al. A High-Affinity Calmodulin-Binding Site in the CyaA Toxin Translocation Domain is Essential for Invasion of Eukaryotic Cells. *Adv. Sci.* **2021**, 8, 2003630.
2. O’Brien, D. P., Cannella, S. E., Voegele, A., Raoux-Barbot, D., Davi, M., Douche, T., Matondo, M., Brier, S., Ladant, D., Chenal, A. Post-translational acylation controls the folding and functions of the CyaA RTX toxin. *FASEB J.* **2019**, 33, fj201802442RR.
3. O’Brien, D. P., Durand, D., Voegele, A., Hourdel, V., Davi, M., Chamot-Rooke, J., Vachette, P., Brier, S., Ladant, D., Chenal, A. Calmodulin fishing with a structurally disordered bait triggers CyaA catalysis. *PLoS Biol.* **2017**, 15, e2004486.
4. Voegele, A., Subrini, O., Sapay, N., Ladant, D., Chenal, A. Membrane-Active Properties of an Amphitropic Peptide from the CyaA Toxin Translocation Region. *Toxins* **2017**, 9, 369.

### 5.7. Is the Regulation of Toxinogenesis the Same in Clostridium botulinum and Clostridium tetani?

Diana Chapeton-Montes ^1^, Holger Brüggemann ^2^, Michel R. Popoff ^1,^*

^1^ Bacterial Toxins, Institut Pasteur, Paris, France.

^2^ Department of Biomedicine, Aarhus University, Aarhus, Denmark.

* Correspondence: popoff2m@gmail.com

**Abstract:** Botulinum neurotoxins (BoNTs) and tetanus neurotoxin (TeNT) share similar structures and molecular modes of action, albeit BoNTs target motor-neuron endings leading to a flaccid paralysis (botulism) and TeNT interacts with central inhibitory interneurons causing spastic paralysis (tetanus). BoNTs and TeNT are produced by anaerobic and sporulating bacteria of the *Clostridium* genus, *C. botulinum* and *C. tetani*, respectively. The synthesis of BoNTs and TeNT is a highly regulated process. Among the diverse *C. botulinum* types, toxinogenesis was mainly investigated in *C. botulinum* A. In both *C. botulinum* A and *C. tetani*, a gene coding for an alternative sigma factor lies upstream of the neurotoxin gene and is required for the expression of the toxin gene. *C. botulinum* and *C. tetani* contain numerous two-component systems (TCSs) (39 in *C. botulinum* A and 30 in *C. tetani*), which are major regulatory systems in response to environmental conditions in prokaryotes. Albeit most TCS genes are homologous in both species, *C. botulinum* A and *C. tetani* use distinct sets of TCSs in the regulation of toxin synthesis. Only one TCS, which is homologous in *C. botulinum* A and *C. tetani*, has a similar function of negative regulation of toxin synthesis in both species. In contrast to *C. botulinum* A, inorganic phosphate and carbonate are environmental factors controlling toxin synthesis in *C. tetani.* In addition, a non-coding RNA downstream the *tent* gene negatively regulates TeNT synthesis. No homologous RNA sequence was found in *C. botulinum* A. Thereby, *C. botulinum* and *C. tetani* are two environmental bacteria that use distinct regulatory pathways to synthesize potent neurotoxins.

**Keywords:** *Clostridium botulinum*; *Clostridium tetani*; botulinum neurotoxin; tetanus neurotoxin; toxin synthesis regulation

**References**

1. Connan, C.; Brueggemann, H.; Mazuet, C.; Raffestin, S.; Cayet, N.; Popoff, M.R. Two-component systems are involved in the regulation of Botulinum neurotoxin synthesis in *Clostridium botulinum* type A strain hall. *PLoS ONE* **2012**, *7*, e41848.
2. Chapeton-Montes, D.; Plourde, L.; Deneve, C.; Garnier, D.; Barbirato, F.; Colombié, V.; Demay, S.; Haustant, G.; Gorgette, O.; Schmitt, C.; et al. Tetanus toxin synthesis is under the control of a complex network of regulatory genes in *Clostridium tetani*. *Toxins* **2020**, *12*, 328.
3. Brüggemann, H.; Chapeton-Montes, D.; Plourde, L.; Popoff, M.R. Identification of a non-coding RNA and its putative involvement in the regulation of tetanus toxin synthesis in *Clostridium tetani*. *Sci. Rep.* **2021**, *11*, 4157.

### 5.8. In Vivo Spatiotemporal Control of Voltage-Gated Ion Channels by Engineered Photoactivatable Peptidic Toxins

Jérôme Montnach ^1,2^, Laila Ananda Blömer ^2,3^, Ludivine Lopez ^1,2,4^, Luiza Filipis ^2,3^, Hervé Meudal ^5^, Aude Lafoux ^6^, Sébastien Nicolas ^1,2^, Duong Chu ^7^, Cécile Caumes ^4^, Rémy Béroud ^4^, Chris Jopling ^8^, Frank Bosmans ^9^, Corinne Huchet ^6^, Céline Landon ^5^, Marco Canepari ^2,3^, Michel De Waard ^1,2,4,^*

^1^ L’institut du thorax, INSERM, CNRS, UNIV NANTES, F-44007 Nantes, France.

^2^ Laboratory of Excellence Ion Channels, Science & Therapeutics, F-06560 Valbonne, France.

^3^ Laboratoire Interdisciplinaire de Physique, Université Grenoble Alpes, CNRS UMR 5588, 38402 Saint Martin-d’Hères cedex, France.

^4^ Smartox Biotechnology, 6 rue des Platanes, F-38120 Saint-Egrève, France.

^5^ Center for Molecular Biophysics, CNRS, rue Charles Sadron, CS 80054, Orléans 45071, France.

^6^ Therassay Platform, IRS2-Université de Nantes, Nantes, France.

^7^ Queen’s University Faculty of Medicine, Kingston, ON, Canada.

^8^ Institut de Génomique Fonctionnelle, 141 rue de la Cardonille, 34094 Montpellier, France.

^9^ Department of Basic and Applied Medical Sciences, Ghent University, Ghent, Belgium.

* Correspondence: michel.dewaard@univ-nantes.fr

**Abstract:** Photoactivatable drugs targeting ligand-gated ion channels opened up new opportunities for light-guided therapeutic interventions. Photoactivable toxins targeting ion channels have the potential to control excitable cell activities with low invasiveness and high spatiotemporal precision. We developed caged HwTxIV-Nvoc, a UV light-cleavable and photoactivatable peptide that targets voltage-gated sodium (Na_V_) channels. We first validated physico-chemical parameters of photolysis, and by using a high-throughput patch-clamp system (SyncroPatch364, Nanion), we validated the activity and photosensitivity of caged HwTxIV-Nvoc in vitro in HEK293 cells. We further pursued our investigations in ex vivo brain slices and in vivo on mice neuromuscular junctions and zebrafish models. We found that caged HwTxIV-Nvoc enables precise spatiotemporal control of neuronal Na_V_ channel function under all conditions tested. We developed multiple photoactivatable toxins and therefore demonstrated the broad applicability of the toxin-photoactivation technology as a tool for experiments but also as a relevant clinical approach in disease management.

**Keywords:** ion channel; photopharmacology; toxin

### 5.9. Functional Impact of BeKm-1, a High-Affinity hERG Blocker, on Cardiomyocytes Derived from Human-Induced Pluripotent Stem Cells

Stephan De Waard ^1,2,#^, Jérôme Montnach ^1,#^, Barbara Ribeiro ^1^, Sébastien Nicolas ^1^, Virginie Forest ^1^, Flavien Charpentier ^1^, Matteo Elia Mangoni ^2,3^, Nathalie Gaborit ^1^, Michel Ronjat ^1,2^, Gildas Loussouarn ^1^, Patricia Lemarchand ^1^, Michel De Waard ^1,2,4,^*

^1^ L’institut du thorax, INSERM, CNRS, Université de Nantes, F-44007 Nantes, France.

^2^ LabEx Ion Channels, Science & Therapeutics, F-06560 Valbonne, France.

^3^ Institut de Génomique Fonctionnelle, CNRS, INSERM, Université de Montpellier, F34094 Montpellier, France.

^4^ Smartox Biotechnology, 6 rue des Platanes, F-38120 Saint-Egrève, France.

* Correspondence: michel.dewaard@univ-nantes.fr

**Abstract:** IKr current, a major component of cardiac repolarization, is mediated by human Ether-*à*-go-go-Related Gene (hERG, Kv11.1) potassium channels. The blockage of these channels by pharmacological compounds is associated with drug-induced long QT syndrome (LQTS), which is a life-threatening disorder characterized by ventricular arrhythmias and defects in cardiac repolarization that can be illustrated using cardiomyocytes derived from human-induced pluripotent stem cells (hiPS-CMs). This study was meant to assess the modification in hiPS-CMs excitability and contractile properties by BeKm-1, a natural scorpion venom peptide that selectively interacts with the extracellular face of hERG, by opposition to reference compounds that act onto the intracellular face. By using an automated patch-clamp system, we compared the affinity of BeKm-1 for hERG channels with some reference compounds. We fully assessed its effects on the electrophysiological, calcium handling and beating properties of hiPS-CMs. By delaying cardiomyocyte repolarization, the peptide induces early afterdepolarizations and reduces spontaneous action potentials, calcium transients and contraction frequencies, therefore recapitulating several of the critical phenotype features associated with arrhythmic risk in drug-induced LQTS. BeKm-1 exemplifies an interesting reference compound in the integrated hiPS-CMs cell model for all drugs that may block the hERG channel from the outer face. Being an easily modifiable peptide, it will serve as an ideal molecular platform for the design of new hERG modulators displaying additional functionalities.

**Keywords:** BeKm-1; hERG; hiPS-cardiomyocyte; LQTS

### 5.10. An Agonist of the CXCR4 Receptor Is Therapeutic for the Neuroparalysis Induced by Bungarus Snake Envenoming

Marco Stazi ^1,^*, Federico Fabris ^1^, Kae Yi Tan ^2^, Aram Megighian ^1^, Alessandro Rubini ^1^, Andrea Mattarei ^3^, Samuele Negro ^1^, Giorgia D’Este ^1^, Florigio Lista ^4^, Ornella Rossetto ^1^, Choo Hock Tan ^5^, Cesare Montecucco ^1,6^

^1^ Department of Biomedical Sciences, University of Padova, Via Ugo Bassi 58/B, Padova 35131, Italy.

^2^ Department of Molecular Medicine, Faculty of Medicine, University of Malaya, Kuala Lumpur 50603, Malaysia.

^3^ Department of Pharmaceutical and Pharmacological Sciences, University of Padova, Padova 35131, Italy.

^4^ Center of Medical and Veterinary Research of the Ministry of Defense, Policlinico Militare, Via Santo Stefano Rotondo 4, Rome 00184, Italy.

^5^ Department of Pharmacology, Faculty of Medicine, University of Malaya, Kuala Lumpur 50603, Malaysia.

^6^ CNR Institute of Neuroscience, Padova 35131, Italy.

* Correspondence: marco.stazi@studenti.unipd.it

**Abstract:** Snake envenoming is a major but neglected human tropical disease. Among venomous snakes, kraits (snakes of the *Bungarus* genus) are medically important venomous species that cause reversible peripheral neuroparalysis. Hospitalization and use of antivenoms derived from an animal immunized with *Bungarus* venoms are the primary therapies that prevent death from early-onset respiratory paralysis. There is a general consensus that additional and non-expensive treatments, which can be delivered even long after the snakebite, are needed. Traumatic or toxic degeneration of peripheral motor neurons, with ensuing neuroparalysis, is characterized by the activation of a pro-regenerative intercellular signaling program. A major player is the intercellular signaling axis consisting of the chemokine CXCL12a, produced by perisynaptic Schwann cells and acting on the CXCR4 receptor expressed on the damaged neuronal axons. The CXCR4 agonist NUCC-390 was recently found to promote axonal growth. Here, we tested its efficacy on the neuroparalysis induced by the venoms of three major krait species, i.e., *Bungarus caeruleus*, *B. multicinctus* and *B. candidus* that are prevalent in Asia. These venoms cause a complete degeneration of motor axon terminals. Functional recovery of the neuromuscular junction was assessed by electrophysiological recordings and by imaging. We report that NUCC-390 administration to venom-injected mice greatly accelerates the recovery from paralysis. These data candidate NUCC-390 to be tested as novel therapeutics to reduce death by respiratory deficits and to improve the recovery of normal neuromuscular physiology, thus reducing the human and hospital costs of envenoming. NUCC-390 can be administered any time after the snakebite and has great potential of becoming a non-expensive addition to the currently available antivenom treatments whose efficacy is limited to a short period after snakebite. This drug is expected to decrease the long and expensive post-snakebite mechanical ventilation phase.

**Keywords:** *Bungarus* venom; CXCR4 agonist; neuromuscular junction

### 5.11. Neutralization of Crotamine by Polyclonal Antibodies against Two Rattlesnake Venoms and a Novel Recombinant Fusion Protein

Roberto Ponce-López ^1^, Alejandro Olvera-Rodríguez ^1,^*, Miguel Borja-Jiménez ^2^, Edgar Neri-Castro ^1^, Leticia Olvera-Rodríguez ^1^, Alejandro Alagón ^1^

^1^ Instituto de Biotecnología, Universidad Nacional Autónoma de México, Cuernavaca, Morelos, México.

^2^ Facultad de Ciencias Biológicas, Universidad Juárez del Estado de Durango, Gómez Palacio, Durango, México.

* Correspondence: alejandro.olvera@ibt.unam.mx

**Abstract:** Current pit-viper antivenoms for human use in Mexico have shown a low level of protection against one neurotoxin found in some rattlesnakes (*Crotalus* spp.), crotamine. This toxin has a low molecular weight (~5 kDa) and is well known for its spastic paralysis symptom provoked in mice with no evidence of neutralization. Recently, it was reported that crotamine is the major toxin found in some rattlesnake venoms such as *C. molossus nigrescens* (~50%) and *C. oreganus helleri* (62%). On the other hand, sphingomyelinase D (SMD) is a highly immunogenic enzyme (MW: ~30 kDa) found in *Loxosceles* spp. spider venom. In this study, we aimed to neutralize the crotamine-induced main symptom of paralysis by employing, as immunogens, two rattlesnake venoms and a novel recombinant fusion protein made from crotamine and SMD, used as a carrier. **Methods**—Crotamine cDNA was synthetized from venom gland mRNA of a *C. m. nigrescens* individual from Mexico, while one plasmid containing *L. reclusa* SMD was available in the lab. We cloned the sphingomyelinase D and crotamine in tandem into the expression vector pQE30 to transform competent Origami *Escherichia coli* cells for the production of the protein. By using the recombinant protein and the whole venoms of *C. o. helleri* and *C. m. nigrescens*, we performed separate immunization protocols in rabbits to measure the antibody recognition to crotamine (ELISA and Western blot) and the neutralization capacity in mice against the main toxin-induced paralysis symptom. **Results**—The fusion protein was obtained at ~10 mg/L of bacterial culture with the expected 37.5 kDa molecular mass as analyzed by SDS-PAGE and Western-blot. The recombinant protein and the two whole crotamine-enriched venoms generated antibodies with cross-reactivity against crotamine from up to seven species. The three experimental antivenoms were able to neutralize the paralysis symptom provoked by crotamine in the mice model. **Discussion/Conclusion**—We showed, for the first time, the neutralization of the crotamine-induced spastic paralysis symptom by three experimental antivenoms. The recombinant SMD-Crotamine fusion protein as well as crotamine-enriched venoms could be good candidate immunogens for the improvement of Mexican antivenoms.

**Keywords:** antivenom; crotamine; rattlesnake; sphingomyelinase-D

**Acknowledgments:** Fordecyt 303045.

### 5.12. Anti-Cancer Effect of the Moroccan cobra (Naja haje) Venom and Its Fractions against Hepatocellular Carcinoma in 3D Cell Culture

Ayoub Lafnoune ^1,2,^*, Su-Yeon Lee ^3^, Jin-Yeong Heo ^4^, Salma Chakir ^1,5^, Khadija Daoudi ^1,2^, Bouchra Darkaoui ^1,2^, Aziz Hmyene ^5^, Rachida Cadi ^2^, Khadija Mounaji ^2^, David Shum ^4^, Haeng-Ran Seo ^3^, Naoual Oukkache ^1^

^1^ Laboratoire des Venins et Toxines, Département de Recherche, Institut Pasteur du Maroc, 1 place Louis Pasteur, Casablanca 20360, Morocco.

^2^ Laboratoire Physiopathologie, Génétique Moléculaire & Biotechnologie, Faculté des Sciences Ain-Chock, Hassan II University of Casablanca, B.P 5366 Maarif, Casablanca 20000, Morocco.

^3^ Cancer Biology Research Laboratory, Institut Pasteur Korea, 16, Daewangpangyo-ro 712 beon-gil, Bundang-gu, Seongnam-si, Gyeonggi-do, 13488 Rep. of Korea.

^4^ Screening Discovery Platform, Institut Pasteur Korea, 16, Daewangpangyo-ro 712 beon-gil, Bundang-gu, Seongnam-si, Gyeonggi-do, 13488 Rep. of Korea.

^5^ Laboratoire de Biochimie Environnement et Agroalimentaire, Faculté des Sciences et Techniques de Mohammedia, Mohammedia, Morocco.

* Correspondence: ayoublafnoune@gmail.com

**Abstract:** Hepatocellular carcinoma (HCC) is the most common primary liver cancer in adults, the fifth most common malignancy worldwide and the third leading cause of cancer-related death. An alternative to the surgical treatments and drugs, such as sorafenib, commonly used in medicine, is necessary to overcome this public health problem. In this study, we determine the anticancer effect on HCC of Moroccan cobra *Naja haje* venom and its fraction obtained by gel filtration chromatography against Huh7.5 cancer cell line. Cells were grown together with WI38 human fibroblast cells, LX2 human hepatic stellate cell line, and human endothelial cells (HUVEC) in MCTS (multi-cellular tumor spheroids) models. The hepatotoxicity of venom and its fractions were also evaluated using the normal hepatocytes cell line (Fa2N-4 cells). Our results showed that an anti HCC activity of *N. haje* venom and, more specifically, the F7 fraction of gel filtration chromatography exhibited the greatest anti-hepatocellular carcinoma effect by decreasing the size of MCTS. This effect is associated with low toxicity against normal hepatocytes. These results strongly suggest that the F7 fraction of *N. haje* venom obtained by gel filtration chromatography possesses the ability to inhibit cancer cell proliferation. More research is needed to identify the specific molecule(s) responsible for the anticancer effect and investigate their mechanism of action.

**Keywords:** anticancer molecule; hepatocellular carcinoma; multicellular tumor spheroid; *Naja haje*; venom

## 6. Poster Presentations (When More than One Author, the Underlined Name Is That of the Presenter)

### 6.1. Circadian Variations in Inflammatory Response and Oxidative Stress during Experimental Scorpion Envenomation

Fares Daachi, Sonia Adi-Bessalem *, Amal Megdad-Lamraoui, Fatima Laraba-Djebari

University of Science and Technology Houari Boumediene (USTHB), Faculty of Biological Sciences, Laboratory of Cellular and Molecular Biology, BP 32 El-Alia, Bab Ezzouar, Algiers, Algeria.

* Correspondence: soniabessalem@hotmail.com

**Abstract:** The mammalian circadian clock orchestrates diverse physiological processes by synchronizing with the nervous and cardiovascular systems, immune response, and metabolic homeostasis. Little is known about the circadian rhythm in the immune response in vivo settings during envenomation—few studies have demonstrated that a circadian pattern might exist. The aim of this study is to investigate whether circadian rhythm affects the inflammation and oxidative stress in envenomed model. In this study, the systemic inflammatory response is induced by the administration of a sublethal dose of *Androctonus australis hector* (Aah) venom to mice during light (1 HALO) and dark (18 HALO) periods. The hypothalamic–pituitary–adrenal (HPA) axis activity was evaluated by measurement of adrenocorticotropic (ACTH) and corticosterone plasma hormones levels as well as by analysis of CD68 immunohistochemistry staining. The inflammation was assessed by the measurement of the serum level of the proinflammatory cytokines, IL-17 and IL-6, evaluation of neutrophil infiltration and oxidative/nitrosative stress markers (Nitrites, MDA, H2O2, CAT and GSH). Glucose level and activities of AST and ALT as well as histopathological analysis of hepatic tissue, were also performed in the two groups of animals (1 HALO and 18 HALO). Obtained results showed a high increase of serum ACTH and corticosterone levels as well as positive immunostaining of CD68 cells during the dark period indicating activation of the HPA axis associated with local inflammation. The results also showed day-night variations, with significantly high levels of nitrite, hydrogen peroxide, myeloperoxidase and lipid peroxidation during the daytime compared with the nighttime. Significant variations in catalase activity and GSH levels are also observed with the highest evening values. Furthermore, an increase in IL-6 levels was observed during the active phase, while no differences were observed in the IL-17 levels between day and night times. These results indicate also that Aah venom induced a time-dependent increase of metabolic parameters during the dark phase and severe hepatocellular injury. In conclusion, the daily variation of the HPA axis activation and inflammatory response appears to be closely related to the circadian moment of envenomation. Future studies should investigate the molecular mechanisms resulting in this circadian rhythmicity.

**Keywords:** circadian variation; inflammation; oxidative stress; scorpion envenomation

### 6.2. Synthesis and Characterization of µ-Conotoxins as Molecular Probes for the Detection of Voltage-Gated Sodium Channel Pore Blockers

Rómulo Aráoz ^1,2,^*, Giovanny Covaleda-Cortés ^1^, Denis Servent ^1,2^

^1^ Université Paris Saclay, CEA, INRAE, Département Médicaments et Technologies pour la Santé (DMTS), SIMoS, 91191 Gif-sur-Yvette, France.

^2^ CNRS, ERL9004, 91191 Gif-sur-Yvette, France.

* Correspondence: romulo.araoz@cea.fr

**Abstract:** Voltage-gated sodium channels (Na_v_) play critical functional roles by controlling, in excitable and non-excitable cells, the action potential initiation/propagation, cell motility, and proliferation. In humans, there are nine subtypes of Na_v_ channels—1.1 to 1.9—with Na_v_1.4 being the muscle subtype. Na_v_ channel dysfunctions are associated with neurological, cardiovascular, muscular and psychiatric disorders. Marine phytoplankton and freshwater cyanobacteria are able to synthesize a series of neurotoxins targeting Na_v_ channels, among them, saxitoxin, a potent site-1 pore blocker classified as a chemical bioweapon because of its high lethality for humans. Receptor binding assays are suitable analytical techniques for ligand screening owing to their extremely high resolution and sensitivity, their fast analysis and easy automation, which facilitates the development of high-throughput protocols. There is, however, a lack of toxin-tracers for Na_v_ toxins detection. The marine cone snail of the genus *Conus* produces a series of conotoxins for prey hunting, among them, μ-conotoxin peptides, with an exquisite affinity for Na_v_ channels. Wild-type and biotinylated synthesis of five μ-conotoxins was performed using a Protein Technologies Prelude synthesizer. Biotinylation was performed on-column after Fmoc deprotection of the N-terminal residue of the μ-conotoxins. After refolding, the resulting conopeptides were purified by reverse-phase HPLC. The affinity of each μ-conotoxin for the Na_v_1.4 channel was determined. Two biotinylated μ-conopeptides with high affinity for Na_v_ channels were identified as potential toxin tracers for the detection of site-1 Na_v_ pore blockers by ligand binding assay.

**Keywords:** cyanotoxin; ligand-binding assay; µ-conopeptide; phycoyoxin; sodium channel

**Acknowledgments:** This research was supported by the NRBC-E Program project MULTITOX (Fiche N° H35 to RA). The authors acknowledge the INTERREG Atlantic Area (ALERTOX-NET EAPA_317/2016 project to DS), and the LABEX LERMIT (DETECTNEUROTOX project, CDE 2017–001173—RD 91 to RA)

### 6.3. Multi-Scale Evaluation of Spider Toxins as Potential Anti-Nociceptive Agents: Example of Cyriotoxin-1a

Evelyne Benoit ^1,^*, Michel De Waard ^2^, Rémy Béroud ^3^, Michel Partiseti ^4^, Denis Servent ^1^

^1^ Service d’Ingénierie Moléculaire pour la Santé, ERL CNRS/CEA 9004, Gif-sur-Yvette, France.

^2^ L’institut du thorax, INSERM UMR 1087/CNRS UMR 6291, Nantes, France.

^3^ Smartox Biotechnology, Saint-Egrève, France.

^4^ Sanofi R & D, Integrated Drug Discovery—High Content Biology, Vitry-sur-Seine, France.

* Correspondence: evelyne.benoit@cea.fr

**Abstract:** Our expertise is the identification and structural and functional characterization of original natural toxins targeting receptors and ion channels, which may have applications in human health. In this context, and in collaboration with *L’institut du thorax*, Smartox Biotechnology and Sanofi R & D, we are interested in the multi-scale evaluation (from the cell in vitro to the organism in vivo) of spider toxins as potential anti-nociceptive agents, as illustrated by the example of Cyriotoxin-1a (CyrTx-1a). Na_V_1.7 channel subtype is highly expressed in the dorsal root ganglia (DRG) of the sensory nervous system and plays a central role in the pain signaling process. We investigated a library prepared from original venoms of 117 different animals to identify new selective inhibitors of this target. We used high-throughput screening of the venom library, using automated patch-clamp experiments on human voltage-gated sodium channel subtypes, and then in vitro and in vivo electrophysiological experiments to characterize the active peptides that were purified, sequenced and chemically synthesized. Analgesic effects were evaluated in mice in vivo. We identified and further characterized CyrTx-1a, a novel peptide isolated from *Cyriopagopus schioedtei* spider venom. This 33 amino acids toxin belongs to the inhibitor cystine knot structural family and inhibits hNa_V_1.1–1.3 and 1.6–1.7 in the low nanomolar range, compared to the micromolar range for hNa_V_1.4–1.5 and 1.8. CyrTx-1a was 920 times more efficient at inhibiting tetrodotoxin (TTX)-sensitive than TTX-resistant sodium currents recorded from adult mouse DRG neurons in vitro and approximately 170 times less efficient than huwentoxin-IV at altering mouse skeletal neuromuscular excitability in vivo. CyrTx-1a exhibited an analgesic effect in mice by significantly increasing reaction time in the hot-plate assay.

**Keywords:** anti-nociceptive toxin; mouse dorsal root ganglia neuron; mouse tactile and hot pain sensitivity; pain; voltage-gated sodium channel subtype

### 6.4. Imaging of Muscular Nicotinic Receptor Distribution Using a Synthetic Fluorescent Analogue of α-Bungarotoxin

Oliver Brun ^1,5,6,^*, Claude Zoukimian ^3^, Barbara Ribeiro De Oliveira Mendes ^1^, Jérôme Montnach ^1^, Michel Ronjat ^1,2^, Rémy Béroud ^3^, Didier Boturyn ^4^, Frédéric Lesage ^5,7^, Michel De Waard ^1,2,3^

^1^ L’institut du thorax, INSERM, CNRS, UNIV NANTES, Nantes, France.

^2^ LabEx “Ion Channels, Science & Therapeutics”, Valbonne, France.

^3^ Smartox Biotechnology, 6 rue des Platanes, F-38120 Saint-Egrève, France.

^4^ Department of Molecular Chemistry, Univ. Grenoble Alpes, CNRS, 570 rue de la chimie, CS 40700, Grenoble 38000, France.

^5^ Research Center, Montreal Heart Institute, Montreal, QC, Canada.

^6^ Faculty of Medicine, Université de Montréal, Montreal, QC, Canada.

^7^ Department of Electrical Engineering, École Polytechnique de Montréal, Montréal, QC, Canada.

* Correspondence: oliver.brun@univ-nantes.fr

**Abstract:** Characterizing the distribution of drug receptors through optical imaging can be challenging, as the availability of suitable fluorescent probes is limited. Over history, natural toxins have benefited science through an ever-growing source of biologically active peptides, many of which show great potential as probes to study the distribution of their associated receptors. Here, we aim at developing a synthetic, fluorescent analog of α-bungarotoxin (α-BgTx), a highly potent, selective and irreversible inhibitor of the muscle-type nicotinic receptors (mnAChR), through the addition of a Cy5 tag. We first performed functional validation of this Cy5-α-BgTx analog by high-throughput patch-clamp (SyncroPatch364, Nanion), looking at the inhibition of acetylcholine-mediated currents on TE671 cells. It, therefore, appeared that the addition of the Cy5 tag still generates a peptide with nanomolar affinity. In order to further ensure that natural binding properties of the peptide were preserved, we synthesized a peptide analog of the α-BgTx binding site on the mnAChR (BSpep). Preincubation of Cy5-α-BgTx with BSpep resulted in the capture of the α-BgTx and complete blockage of its inhibitory effect. The fluorescent toxin was then used to study the distribution of mnAChR on fixed TE671 cells, allowing us to visualize both its intra and extracellular distribution. Thus, we were able to synthesize a functional, synthetic analog of α-BgTx that can be used to localize mnAChRs, highlighting the potential of toxin-derived biopeptide engineering as a tool for receptor imaging.

**Keywords:** α-bungarotoxin; peptide engineering; receptor imaging

### 6.5. Proteomic Analysis of the Predatory Venom of Conus striatus Reveals Population-Specific Glycosylation Patterns in Kappa A-Conotoxins

Claudia Murraciole-Bich, Sébastien Dutertre *

Institut des Biomolécules Max Mousseron (IBMM), Université de Montpellier, CNRS, ENSCM, Montpellier, France.

* Correspondence: sebastien.dutertre@umontpellier.fr

**Abstract:** Animal venoms are a rich source of pharmacological compounds with ecological and evolutionary significance, as well as with therapeutic and biotechnological potentials. Among the most promising venomous animals, cone snails produce potent neurotoxic venom to facilitate prey capture and defend against aggressors. The main objective of this study was to investigate and decipher the composition of the predatory venom injected by *Conus striatus*, one of the largest and most widely distributed piscivorous species. Standard LC-MS analysis provided an overview of native venom compounds, whereas LC-MS/MS followed by bioinformatic analysis identified many conotoxin sequences. Kappa A-conotoxins, which contain three disulfide bridges and complex glycosylation, are by far the dominant compounds in predatory venom. Remarkably, different and population-specific glycosylation patterns were detected in specimens from the Pacific and Indian oceans, providing a new level of intraspecific variations for the first time.

**Keywords:** conotoxin; *Conus striatus*; glycosylation; KappA-conotoxin

### 6.6. Why Is Androctonus australis Venom from Far more Studied Than A. mauritanicus One? Does the Answer Reside in the Venom Composition?

Lou Freuville ^1,^*, Fernanda Gobbi Amorim ^1^, Alain Brans ^2^, Rudy Fourmy ^3^, Aude Violette ^3^, Loïc Quinton ^1^

^1^ Laboratory of Mass Spectrometry, MolSys Research Unit, University of Liège, Liège, Belgium.

^2^ Centre for Protein Engineering, University of Liège, B 4000 Liège, Belgium.

^3^ Alphabiotoxine Laboratory sprl, Barberie 15, Montroeul-au-bois 7911 Belgium.

* Correspondence: lfreuville@uliege.be

**Abstract:** Scorpions are characterized by the great complexity and diversity of their venom, which has evolved over 400 million years into a well-elaborated library of powerful toxins mostly targeting, with high specificity, ion channels. Moreover, protein or non-protein compounds found in such venoms display antibacterial, antiviral or again anticancer activity, converting them from toxic molecules to therapeutic and benefic ones. Even if scorpions constitute a target of choice for many researchers, the high number of identified species (>2000) imposes that some of them remain little-known. It is even still the case for the Buthidae family, which constitutes the most human medically relevant species, due to the high concentration of potent ion-channel toxins in their venoms. As an example, despite their phylogenetical proximity, *Androctonus australis* is the heart of 365 publications (Pubmed), whereas *A. mauritanicus* is only considered in five of them. In this work, we aim at comparing the venom profiles of these scorpions at the protein level. A first macroscopic overview of the protein content of each venom was evaluated by 1D SDS-PAGE and showed no notable difference. During the second time, the crude venoms of the two scorpions were analyzed with bottom-up proteomics. Toxins were classically reduced to remove the disulfide bonds, alkylated to avoid their reformation and finally digested with trypsin. The LC-MS/MS spectra were realized with M-Class UPLC coupled to a Q-Exactive spectrometer. Data analysis was performed with the bioinformatics dedicated software “Peaks X+”, with the use of an extracted database from Uniprot built with the keyword “Buthidae”. As preliminary results, *A. mauritanicus* presents 163 Top proteins against 142 for *A. australis* with this database. They share 100 peptides and 63 proteins in common. A table with the comparison of the classification of proteins in each species is presented below (Table 1). In the light of the protein classification, some groups of non-toxins are unexplored in the scorpion venoms, such as the Kunitz-type family and Cysteine-rich venom proteins. Therefore, the proteomic approaches can still open perspectives for the study of these groups, which is relevant to highlight them for future explorations. Comparing a well-studied scorpion species with another less studied but phylogenetically close is interesting to highlight the evolution, diversity and complexity of venom.

**Keywords:***Androctonus mauritanicus*; LC-MS/MS; scorpion venom

### 6.7. New Proteomic Approach for Inventorying the Snake Venom Arsenal

Fernanda Gobbi Amorim ^1,^*, Damien Redureau ^1^, Nicholas Casewell ^2^, Loïc Quinton ^1^

^1^ Laboratory of Mass Spectrometry, MolSys Research Unit, University of Liège, Liège, Belgium.

^2^ Centre for Snakebite Research and Interventions, Liverpool School of Tropical Medicine, Pembroke Place, Liverpool, UK.

* Correspondence: fgamorim@uliege.be

**Abstract:** Multi-Enzymatic Limited Digestion (MELD) is a new methodology that applies synergic and time-limited digestion of multiple enzymes, representing a versatile yet straightforward approach for a new generation of proteomics methodology (MORSA et al., 2019). However, until now, this approach has not been applied to venomic studies, which may be useful to define the venom composition. The generation of a higher number of peptides per protein during the MELD digestion increases the quality of protein sequencing and, consequently, identification. Herein we applied the MELD strategy for the venomics of two snake species: *Echis ocellatus* (EoV) and *Dendroaspis polylepis* (DpV). Ten micrograms of each venom were reduced/alkylated followed by two different digestion protocols: (1) trypsin and (2) MELD (Trypsin, GluC and Chymotrypsin). The digested materials were analyzed in a Q-Exactive™ Plus Mass Spectrometer with protein identification performed by Peaks Studio X+ using the Uniprot and transcriptomic databases. MELD showed more peptides/proteins identified for both venoms compared to the trypsin protocol. In EoV, 82.8% were identified as toxins and 17.2% as non-toxins, compared to the trypsin protocol, which resulted in 69.2% toxins, 24% non-toxins and 6.9% for cellular components (CC). In DpV, MELD showed coverage of 26.2% for toxins, 39.9% for non-toxins and 33.9% for CC, while for trypsin, we obtained 23.3% for toxins, 37.4% for non-toxins and 39.3% for CC. MELD was able to identify new components in both venoms. Fifty-one percent of the EoV were metalloproteinases (MP), while DpV showed a high content of nerve growth factor (22%), which was not identified using the transcriptome database. The highest number of mass spectra was obtained for a metalloproteinase (tr|Q2UXQ4) for *E. ocellatus*, in which the MELD approach led to four times more mass spectra. For *D. polylepis*, dendrotoxin I (P00979) showed the highest number of mass spectra, and the trypsin-based approach yields two times more mass spectra. As the number of mass spectra gives a rough evaluation of the toxin concentration, the two cited toxins are among the most produced in the venoms. MELD presented a different coverage according to the presence of high molecular mass content in the venom arsenal. This strategy can be applied to identify new groups of venom components. It represents an innovative strategy for venomics, opening new perspectives for sequencing and inventorying the venom arsenal.

**Keywords:** proteomics; snake; toxin; venom

### 6.8. Nano-Rattlers against Cancer: Snake Venom-Loaded Nanoparticles against Tumoral Cell Lines

Jorge Jimenez-Canale ^1,^*, Nayelli-Guadalupe Teran-Saavedra ^1^, Enrique-Fernando Velazquez-Contreras ^1^, Martin-Samuel Hernandez-Zazueta ^2^, Hector-Manuel Sarabia-Sainz ^3^, Daniel Fernandez-Quiroz ^4^, Alexel-Jesus Burgara-Estrella ^5^, Jose-Andrei Sarabia-Sainz ^5^

^1^ Departamento de Investigacion en Polimeros y Materiales, Universidad de Sonora, blvd. Luis Encinas s/n, CP 83000, Hermosillo, Sonora, Mexico.

^2^ Departamento de Investigacion y Posgrado en Alimentos, Universidad de Sonora, blvd. Luis Encinas s/n, CP 83000, Hermosillo, Sonora, Mexico.

^3^ Departamento de Ciencias del Deporte y de la Actividad Fisica, Universidad de Sonora, blvd. Luis Encinas s/n, CP 83000, Hermosillo, Sonora, Mexico.

^4^ Departamento de Ingenieria Quimica y Metalurgia, Universidad de Sonora, blvd. Luis Encinas s/n, CP 83000, Hermosillo, Sonora, Mexico.

^5^ Departamento Investigacion en Fisica, Universidad de Sonora, blvd. Luis Encinas s/n, CP 83000, Hermosillo, Sonora, Mexico.

* Correspondence: jorgejimzc@gmail.com

**Abstract:** Research in drug delivery systems has led to the development and improvement of materials that affect the pharmaceutical effect of bioactive components, broadening the options of treatment for several diseases, including cancer. Chitosan (Cs) has been firmly established as a biocompatible and biodegradable low-toxic polymer able to form complexes with bioactive agents, making them promising drug delivery vehicles. Additionally, some snake venom toxins such as phospholipases A2 (PLA2s), serine proteinases (SVSPs) and metalloproteinases (SVMPs) were reported to present cytotoxic activity in different tumor cell lines, making them an auspicious option to be used as cancer pharmaceuticals. In the present study, we identified the major proteins in the venom of a northern black-tailed rattlesnake (*Crotalus molossus molossus*) and cytotoxic activity against T-47D breast carcinoma cells were evaluated. Afterward, the venom was loaded into a Cs NPs system through the ionotropic gelation process with tripolyphosphate (TPP), obtaining particles of 415.9 ± 21 nm and zeta potential of +28.3 ± 1.1 mV. The venom-loaded Cs-ALG complex was able to deliver the venom into the breast carcinoma cells through endocytosis, inhibiting their viability and inducing morphological changes. Although more studies are required, we suggest the potential use of *C. m. molossus* venom toxins entrapped within polymer nanoparticles for the future development and research of tumor pharmaceuticals.

**Keywords:** chitosan; nanomedicine; nanoparticle; snake venom

### 6.9. Adenosine Amplifies Granulopoiesis and Systemic Inflammatory Response after Scorpion Envenomation

Asma Kaddache *, Louiza Bechohra, Fatima Laraba-Djebari, Djelila Hammoudi-Triki

Department of Cellular and Molecular Biology, University of Science and Technology Houari Boumediene (USTHB), BP 32 El-Alia, Bab Ezzouar, Algiers, Algeria.

* Correspondence: akaddache@usthb.dz

**Abstract:** Scorpion envenomation (SE) triggers granulopoiesis, resulting in heightened production of neutrophils that, in turn, exacerbate tissue damage. However, the mechanisms that sustain their production and recruitment to the injured tissue are unclear. Experimental evidence suggests that alarmines, inter alia adenosine, can modulate proliferation and differentiation of hematopoietic progenitors as well as mature neutrophils mobilization. Indeed, adenosine accumulates in the extracellular space in response to cell damage and can modulate immune cells through its transmembrane receptors A1, A2A, A2B, and A3. However, despite increasing data incriminating adenosine in many models of inflammation, little is known about its effect in the inflammatory response caused by the venom of *Androctonus australis hector* (Aah). **Aim**—In this study, we focused on deciphering molecular signals that promote neutrophil production and recruitment after SE by targeting adenosine as a potential candidate. **Methods**—Using a mouse model of envenomation and a pharmacological strategy, we prevented adenosine signaling by 1,3,7-triméthylxanthine, a nonselective inhibitor of adenosine A1 and A2A receptors. We first characterized the effect of this treatment on granulopoiesis in the bone marrow and then evaluated the expanse of inflammation to various tissues, mainly of the cardiorespiratory system. To this end, medullar MPO activity was assessed as a marker of progenitor cells proliferation and differentiation. In parallel, differential count of leucocytes and histopathological analysis of heart and lungs were carried out. **Results**—Inhibition of the action of adenosine helped to attenuate venom-induced granulopoiesis and reduced the accumulation of neutrophils in the blood. The damages caused by neutrophils in the lungs and heart were improved to a large extent. **Conclusion**—These results highlight the involvement of adenosine in the inflammatory response induced after SE via its A1 and A2A receptors and could help identify a new target to modulate inflammation and prevent cardiorespiratory failure in envenomed patients.

**Keywords:** adenosine; granulopoiesis; scorpion envenomation

### 6.10. Comparative Study of Two Thrombin-Like Molecules from Vipera lebetina Venom

Amel Kadi-Saci *, Fatima Laraba-Djebari *

University of Science and Technology Houari Boumediene (USTHB), Faculty of Biological Sciences, Laboratory of Cellular and Molecular Biology, BP 32 El-Alia, Bab Ezzouar, Algiers, Algeria.

* Correspondence: amel_saci@yahoo.fr; flaraba@hotmail.com; flaraba@usthb.dz

**Abstract:** Snake venoms contain a wide range of components that are able to initiate or inhibit several steps of coagulation or platelet aggregation. Two thrombin-like enzymes, VLCV and VLCII (45 and 60 kDa, respectively) with fibrinogenolytic and pro-aggregating activities, are purified from *Vipera lebetina* venom. These molecules of interest are involved in platelet adhesion to fibrinogen by activating signaling pathways via αIIbβ3 integrins and thus binding to fibrinogen. Results also showed that both molecules present a high pro-aggregating effect with a similar mechanism of thrombin. The incubation of heparin with VLCV or VLCII has no inhibition on induced platelet aggregation by these molecules, suggesting that both enzymes have similar functional characteristics. The use of PMSF also has no effect on the activity of both molecules, indicating that the induced platelet aggregation by VLCV or VLCII involves an independent mechanism of catalytic activity. Results also indicate that both molecules VLCII and VLCV present a mechanism of action involving the thrombin and/or ADP pathways via PAR1/PAR4/P2Y12 receptors, as well as the αIIbβ3 integrin, leading to adhesion and platelets activation. Both thrombin-like VLCII and VLCV could be used as potential targets for the development of new drugs.

**Keywords:** biomolecule; coagulation; pro-aggregating; snake venom; thrombin-like

### 6.11. A comparative Study of the Histological Changes Induced by the Venoms of the Moroccan Snakes Cerastes cerastes and Naja haje

Soukaina Khourcha ^1,2,^*, Salma Chakir ^1,2^, Ayoub Lafnoune ^1^, Bouchra Darkaoui ^1^, Khadija Daoudi ^1^, Fatima Chgoury ^1^, Abdelaziz Hmyene ^2^, Naoual Oukkache ^1^

^1^ Laboratory of Venoms and Toxins, Institut Pasteur of Morocco, 1 place Louis Pasteur, Casablanca 20360, Morocco.

^2^ Laboratory of Biochemistry, Environment and Food Technology, Faculty of Sciences and Technologies, BP 146, Mohammedia 20650, Morocco.

* Correspondence: khourcha.soukaina9@gmail.com

**Abstract:** In Morocco, ophidian envenomations are perpetrated by eight species of snakes belonging to the families of vipers and elapids, including *Cerastes cerastes* (Cc) and *Naja haje* (Nh), respectively. The present work offers a comparative study between the two venoms from a toxicological and physiopathological standpoint. We first determined the toxicity of the Cc and Nh venoms by an LD50 test and then carried out an anatomopathological study on Swiss mice in order to detect the signs of envenomation by each venom. The organs of the envenomed mice were then removed for a histological study to determine the main systemic alterations induced by the venoms. The results of our comparative study showed large disparities between the two venoms in terms of toxicity, revealing that the Nh venom is more toxic than the Cc venom. This disparity is also evident in the MHD test and the histopathological study that shows that the Nh venom exerts a cytotoxic activity on the brain, heart, lungs, liver and kidneys, while that of Cc leads to the formation of hemorrhagic foci and lesions on the liver, kidneys and heart.

**Keywords:** characterization; histology; physiopathology; snake; toxicity; venom

### 6.12. Modulation of Induced Neuro–Immuno–Inflammatory Response by K^+^ Neurotoxin: Involvement of Serotonin and Histamine Receptors

Amina Ladjel-Mendil ^1,^*, Marie-France Martin-Eauclaire ^2^, Fatima Laraba-Djebari ^1^

^1^ University of Science and Technology Houari Boumediene (USTHB), Faculty of Biological Sciences, Laboratory of Cellular and Molecular Biology, BP 32, El–Alia Bab Ezzouar, 16111 Algiers, Algeria.

^2^ Aix-Marseille University, CRN2M CNRS UMR 7290, IFR Jean-Roche, Faculté de Médecine Secteur Nord, boulevard Pierre Dramard, 13916 Marseille cedex 20, France.

* Correspondence: mendilamina@yahoo.fr

**Abstract:** The neuro–immuno–inflammatory response triggered by neurotoxins is a key event in the pathogenesis of scorpion envenomation. In the present study, this response was evaluated in cardiac, pulmonary and brain tissues of intoxicated mice with kaliotoxin; a neurotoxin derived from *Androctonus australis hector* scorpion venom. The involvement of serotoninergic and histaminergic pathways in the systemic inflammatory response following kaliotoxin administration was also investigated. Obtained results revealed that the injected kaliotoxin by intracerebroventricular route induces an important immune-inflammatory response in brain, cardiac and pulmonary tissues. This response is mainly characterized by local features such as edema formation, inflammatory cell infiltration and imbalanced redox status. These effects are correlated with severe tissue alterations and a concomitant increase in metabolic enzymes in sera. Pretreatment of mice with antagonists of serotonin (5HT) and histamine (H1) receptors markedly attenuated these alterations in all the studied tissues. Serotonin and histamine-specific receptors seem to be the main pharmacological targets involved in the neural and systemic inflammatory processes. These findings could help to understand better the role of serotonin and histamine in scorpion venom-induced inflammatory response and pave the way to new therapies targeting 5HT and H1 receptors in order to attenuate the induced neuro–immuno–inflammatory disturbances that may be encountered in severe grades of scorpion envenoming.

**Keywords:** 5HT and H1 receptors; immunoinflammatory response; kaliotoxin; oxidative stress; tissue injury

### 6.13. High-Throughput Screening of Spider Venoms for Identification of Active Compound in Voltage-Gated Sodium Channel

Ludivine Lopez ^1,2,^*, Jérôme Montnach ^1^, Sébastien Nicolas ^1^, Lucie Jaquillard ^2^, Rémy Béroud ^2^, Michel De Waard ^1^

^1^ L’institut du thorax, INSERM UMR 1087/CNRS UMR 6291, LabEx “Ion Channels, Science & Therapeutics”, F-44007 Nantes, France.

^2^ Smartox Biotechnology, 6 rue des Platanes, F38120 Saint Egrève, France.

* Correspondence: ludivine.lopez@univ-nantes.fr

**Abstract:** Dysfunctions of voltage-gated sodium channels (Nav) have been associated with many pathologies such as cardiac diseases, neuropathic pain and epilepsy. In order to study the role of these channels in diseases or to restore their function, selective molecules targeting ion channels are needed. Only a few molecules selective to a single sodium channel isoform have been discovered. This makes these channel types important targets in drug discovery. Animal venoms, and especially spider venoms, contain various peptides that target ion channels. By screening spider venoms on Nav isoforms, we aimed to identify new peptidic toxins targeting specifically one of them. We performed a screening of about thirty spider venoms on Nav1.3, Nav1.4, Nav1.5 and Nav1.6 isoforms using the automated patch-clamp (APC) technique (SyncroPatch364, Nanion). All venoms were preliminarily fractionated in 64 fractions and tested on each Nav. Fractions of interest are those that reduce sodium peak current (by at least 30%), slow-down inactivation or increase late sodium current. False-positive fractions were excluded based on the detection of material in HPLC or mass spectrometry. The primary screening identified 220 fractions active on at least one isoform. Some of these fractions were then selected for purification and tested again on the automatic patch-clamp (74 for Nav1.3, 42 for Nav1.4, 28 for Nav1.5 and 23 for Nav1.6). The most interesting peptides were sequenced, synthesized and characterized. This study suggests that among a large number of toxins present in venoms, close to 50% of them target sodium channels with specificity for each sodium channel isoform.

**Keywords:** 5HT screening; automated patch-clamp; spider toxin

### 6.14. Gambierol Action on K^+^ Currents and Catecholamine Release in Cultured Single Chromaffin Cells from Fetal Rat Adrenal Medulla

Jordi Molgó ^1,2,^*, Roland Bournaud ^2^, Sébastien Schlumberger ^2^, Makoto Sasaki ^3^, Haruhiko Fuwa ^4^, Denis Servent ^1^, Evelyne Benoit ^1,2^

^1^ Université Paris-Saclay, CEA, INRAE, Institut des sciences du vivant Frédéric Joliot, Département Médicaments et Technologies pour la Santé (DMTS), Service d’Ingénierie Moléculaire pour la Santé (SIMoS), ERL CNRS 9004, F-91191 Gif-sur-Yvette, France.

^2^ CNRS, Laboratoire de Neurobiologie Cellulaire et Moléculaire-UPR 9040, F-91198 Gif-sur-Yvette, France.

^3^ Tohoku University, Graduate School of Life Sciences, Sendai, Japan.

^4^ Chuo University, Faculty of Science and Engineering, Department of Applied Chemistry, 1-13-27 Kasuga, Bunkyo, Tokyo 112-8551, Japan.

* Correspondence: jordi.molgo@cea.fr

**Abstract:** Gambierol, characterized by a transfused octacyclic polyether core, was first isolated and chemically typified from cultured *Gambierdiscus toxicus* dinoflagellates collected in French Polynesia. Subsequently, distinct groups in Japan and the USA, using various strategies, achieved their total chemical synthesis. This allowed detailed studies on its mode of action. Gambierol inhibits voltage-gated K^+^ (K_V_) channels in various excitable and non-excitable cells, as well as in motor nerve terminals of the skeletal neuromuscular junction. In the present study, we investigated first the effects of nanomolar concentrations of gambierol on K^+^ current of cultured chromaffin cells from fetal rat adrenal medulla using perforated patch-clamp current recordings. Our results show that gambierol only blocked a small component of the total K^+^ current and affected neither calcium-activated K^+^ (K_Ca_) nor ATP-sensitive K^+^ (K_ATP_) channels, as revealed using apamin and iberiotoxin (selective K_Ca_ channel blockers) and glibenclamid (K_ATP_ channel blocker). After inhibiting K_ATP_ and K_Ca_ channel activation, the gambierol concentration blocking 50% of the K^+^ current component (IC_50_) was 7.6 ± 1.1 nM. The repolarizing phase of all-or-none elicited action potentials, recorded under current-clamp conditions (triggered by 1-ms depolarizing pulses), was sensitive to the action of gambierol but insensitive to the action of apamin and iberiotoxin, indicating that K_Ca_ channels do not participate in the modulation of action potential duration triggered by short depolarizing pulses. The use of simultaneous patch-clamp and single-cell amperometry allowed controlling the membrane potential and detecting exocytosis events (with a carbon electrode polarized to +650 mV to allow the oxidation of released catecholamines). Such recordings revealed that gambierol did not modify the membrane potential following 14-s depolarizing current steps and did not significantly increase the number of exocytotic catecholamine release events with respect to controls. The addition of K_Ca_ channel blockers (in the continuous presence of gambierol) enhanced the membrane depolarization by about 15 mV (during the 14 s current step), and at the same time, increased significantly the number of exocytotic events related to catecholamine secretion. Such enhanced depolarization induced by the K_Ca_ channel blockers probably brings the membrane potential above the activation threshold of high-voltage activated Ca_V_ channels, triggering both Ca^2+^ influx and subsequent catecholamine secretion. These results emphasize the diversity of K_V_ channels in chromaffin cells from fetal rat adrenal medulla and highlight the modulatory role played by K_Ca_ channels in the control of exocytosis in the absence of splanchnic innervation.

**Keywords:** catecholamine release; cultured chromaffin cell; fetal rat adrenal medulla; gambierol; potassium current

### 6.15. Sprouting and Convergent and Stable Polyinnervation Characterize Human Orbicularis oculi Muscles Treated for Blepharospasm with Repeated Botulinum Type A Neurotoxin Injections

Brigitte Girard ^1^, Aurélie Couesnon ^2^, Émmanuelle Girard ^3^, Jordi Molgó ^2,4,^*

^1^ Service d’Ophtalmologie—Hôpital Tenon, 4 rue de la Chine 75020 Paris, Sorbonne Université, UPMC, France.

^2^ Institut des Neurosciences Paris-Saclay, UMR 9197, CNRS/Université Paris-Sud, 91198 Gif-sur-Yvette cedex, France.

^3^ Institut NeuroMyoGene, CNRS UMR 5310, INSERM U1217, Université Lyon1, 8 avenue Rockefeller, 69008 Lyon, France.

^4^ Université Paris-Saclay, CEA, INRAE, Institut des sciences du vivant Frédéric Joliot, Département Médicaments et Technologies pour la Santé, Service d’Ingénierie Moléculaire pour la Santé, ERL CNRS 9004, F-91191 Gif-sur-Yvette, France.

* Correspondence: jordi.molgo@cea.fr

**Abstract:** Currently, a number of pathological conditions, including movement disorders, are treated with botulinum type-A neurotoxin (BoNT/A). This neurotoxin is known to block quantal acetylcholine (ACh) release from motor nerve terminals of the skeletal neuromuscular junction (NMJ), causing temporary muscle paralysis. The treatment with BoNT/A has shown its clinical efficacy in the management of benign blepharospasm, a form of focal dystonia. Due to the slow functional recovery of neuromuscular transmission after BoNT/A-treatment, patients with blepharospasm receive recurrent (every 2–3 months) intramuscular toxin injections in the affected muscles. Regardless of this limitation, the toxin securely relieves patients from their dystonia symptoms and considerably enriches their quality of life. Nevertheless, in less than 4% of the blepharospasm patients treated at Tenon Hospital, BoNT/A-treatment was no longer effective in relieving the patient’s symptoms, and it was compulsory to perform the upper myectomy of the *Orbicularis oculi* muscle. In this study, we used surgical waste muscle specimens from 14 patients treated with repeated injections of either Dysport^®^, abobotulinumtoxinA Ipsen or Xeomin^®^ incobotulinumtoxinA, Merz. These muscle specimens were compared to others, obtained in normal subjects (naïve of BoNT/A) during blepharoplasty. The morphological study was performed blinded to the BoNT/A sample treatment. Neuromuscular specimens analyzed by confocal laser scanning microscopy, using fluorescent staining and immune-labeling of presynaptic proteins, revealed that the pattern of innervation, the muscle nicotinic ACh receptors (nAChR) and the NMJs exhibited marked differences in BoNT/A-treated muscles (regardless of the toxin clinically used), with respect to controls. The control muscles were constantly innervated by a single motor axon (mono-innervated), and NMJs were relatively simple with the typical nAChR array. In contrast, most of the BoNT/A-treated muscles exhibited profuse and stable poly-neuronal innervation, with two unambiguous features: one in which multiple axons innervated a single muscle fiber, the other in which distinct motor axons converged to a unique endplate (convergent innervation). The ability to increase the proportion of poly-innervated muscle fibers may be related to either the stimulation of nerve sprouting (due to muscle inactivity caused by BoNT/A) or the absence of synapse elimination. During development, the structures composing the NMJ undergo rapid formation and elimination. In rodents, unessential synapses are eliminated. We previously reported synapse elimination in mature rodent NMJs following a single BoNT/A injection. The new findings reported here raise a number of questions about the origin and factors contributing to the plasticity changes observed and the expected detrimental functioning of NMJs and muscle fibers.

**Keywords:** botulinum neurotoxin type A; innervation pattern; sprouting

### 6.16. Synthetic Analogues of Huwentoxin-IV Spider Peptide with Altered Human Na_V_1.7/Na_V_1.6 Selectivity Ratios

Ludivine Lopez ^1^, Jérôme Montnach ^1,^*, Barbara Oliveira-Mendes ^1^, Baptiste Thomas ^2^, Cécile Caumes ^2^, Sébastien Nicolas ^1^, Denis Servent ^3^, Charles Cohen ^4^, Rémy Béroud ^2^, Evelyne Benoit ^3^, Michel De Waard ^1,2,5^

^1^ L’institut du thorax, INSERM, CNRS, UNIV NANTES, Nantes, France.

^2^ Smartox Biotechnology, Saint-Egrève, France.

^3^ Université Paris Saclay, CEA, Institut des sciences du vivant Frédéric Joliot, Département Médicaments et Technologies pour la Santé (DMTS), Service d’Ingénierie Moléculaire pour la Santé (SIMoS), ERL CNRS/CEA 9004, Gif-sur-Yvette, France.

^4^ Xenon Pharmaceuticals, Burnaby, British Columbia, Canad. ^5^ LabEx “Ion Channels, Science and Therapeutics”, Valbonne, France.

* Correspondence: jerome.montnach@univ-nantes.fr

**Abstract:** Huwentoxin-IV (HwTx-IV), a peptide discovered in the venom of the Chinese bird spider *Cyriopagopus schmidti*, has been reported to be a potent antinociceptive compound due to its action on the genetically validated Na_V_1.7 pain target. Using this peptide for antinociceptive applications in vivo suffers from one major drawback, namely its negative impact on the neuromuscular system. Although it was studied only recently, this effect appears to be due to an interaction between the peptide and the Na_V_1.6 channel subtype located at the presynaptic level. The aim of this work was to investigate how HwTx-IV could be modified in order to alter the original human (h) Na_V_1.7/Na_V_1.6 selectivity ratio of 23. Nineteen HwTx-IV analogs were chemically synthesized and tested for their blocking effects on the Na^+^ currents flowing through these two channel subtypes stably expressed in cell lines. The dose–response curves for these analogs were generated, thanks to the use of an automated patch-clamp system. Several key amino acid positions were targeted owing to the information provided by earlier structure–activity relationship (SAR) studies. Among the analogs tested, the potency of HwTx-IV E^4^K was significantly improved for hNa_V_1.6, leading to a decreased hNa_V_1.7/hNa_V_1.6 selectivity ratio (close to 1). Similar decreased selectivity ratios, but with increased potency for both subtypes, were observed for HwTx-IV analogs that combine a substitution at position 4 with a modification of amino acid 1 or 26 (HwTx-IV E^1^G/E^4^G and HwTx-IV E^4^K/R^26^Q). In contrast, increased selectivity ratios (>46) were obtained if the E^4^K mutation was combined to an additional double substitution (R^26^A/Y^33^W) or simply by further substituting the C-terminal amidation of the peptide by a carboxylated motif, linked to a marked loss of potency on hNa_V_1.6 in this latter case. These results demonstrate that it is possible to significantly modulate the selectivity ratio for these two channel subtypes in order to improve the potency of a given analog for hNa_V_1.6 and/or hNa_V_1.7 subtypes. In addition, selective analogs for hNa_V_1.7, possessing better safety profiles, were produced to limit neuromuscular impairments.

**Keywords:** huwentoxin-IV; selectivity; sodium channel

### 6.17. Characterization of a Highly Neutralizing Single Monoclonal Antibody to Botulinum Neurotoxin Type A

Christine Rasetti-Escargueil ^2,^*, Sébastien Brier ^1^, Anne Wijkhuisen ^3^, Stéphanie Simon ^3^, Maud Marechal ^2^, Émmanuel Lemichez ^2^, Michel R. Popoff ^2^

^1^ Biological NMR Technological Plateform, Institut Pasteur, CNRS UMR 3528, 75015 Paris, France.

^2^ Institut Pasteur, Toxines Bactériennes, CNRS ERL6002, 75015 Paris, France.

^3^ Université Paris-Saclay, CEA, INRAE, Département Médicaments et Technologies pour la Santé, 91191 Gif-sur-Yvette, France.

* Correspondence: christine.rasetti-escargueil@pasteur.fr

**Abstract:** Monoclonal antibodies (mAbs) represent an alternative and safer way to treat botulism, a fatal flaccid paralysis due to botulinum neurotoxins (BoNTs). In addition, mAbs offer the advantage of being produced in a reproducible manner. We previously identified a unique and potent mouse mAb (TA12) targeting BoNT/A1 with high affinity and neutralizing activity. **Methods**—We characterized the molecular basis of TA12 neutralization by combining Hydrogen/Deuterium eXchange Mass Spectrometry (HDX-MS) with site-directed mutagenesis and neutralization studies. **Results**—The TA12 recognized a conformational epitope located at the interface between the HCN and HCC subdomains of the BoNT/A1 receptor-binding domain (HC). This interface shares common structural features with the ciA-C2 VHH epitope and lies on the face opposite recognized by ciA-C2 and the CR1/CR2 neutralizing mAbs. The single substitution of N1006 was sufficient to affect TA12 binding to HC, confirming the position of the epitope. We further uncovered that the TA12 epitope overlaps with the BoNT/A1 binding site for both the neuronal receptor synaptic vesicle glycoprotein 2 isoform C (SV2C) and the GT1b ganglioside. In vivo neutralization studies confirmed that TA12 is highly neutralizing the most potent subtype BoNT/A1 with medium neutralizing activity against BoNT/A2 and A3 subtypes and low neutralizing activity against BoNT/A5. **Conclusion**—Hence, TA12 potently blocks the entry of BoNT/A1 into neurons by interfering simultaneously with the binding of SV2C and, to a lower extent, GT1b. Our study reveals the unique neutralization mechanism of TA12 and emphasizes the benefit of single mAbs to treat botulism type A.

**Keywords:** botulinum neurotoxin; epitope; monoclonal antibody

### 6.18. Steatoda nobilis: A Comparative Study including Full-Body MALDI Imaging and Application to the Venomics Field

Damien Redureau ^1^, Mathieu Tiquet ^1^, John P. Dunbar ^2^, Fernanda Gobbi Amorim ^1^, Michel M. Dugon ^2^, Loïc Quinton ^1,^*

^1^ Mass Spectrometry Laboratory, MolSys RU, University of Liège, 4000 Liège, Belgium.

^2^ Venom Systems & Proteomics Lab, School of Natural Sciences, Ryan Institute, National University of Ireland Galway, H91 TK33 Galway, Ireland.

* Correspondence: loic.quinton@uliege.be

**Abstract:** The Noble false widow spider *Steatoda nobilis* is a member of the Theridiidae family, akin to the “true” Black widows of the genus Latrodectus. *S. nobilis* is rapidly expanding its geographic range throughout Europe and parts of the Americas, particularly in and around human dwellings. *S. nobilis* has also been shown to be of medical significance in the UK and in Ireland, where a growing number of severe cases of envenomation have occurred over the past five years [Dunbar, 2021]. The comparison of venom composition of male and female *S. nobilis*, using a proteo-transcriptomic approach, demonstrates that male venom contained a lower quantity and diversity of latrotoxins. In order to illustrate the comparison of both male and female *S. nobilis* profiles, we additionally present the first whole-body imaging of a spider using MALDI mass spectrometry. This proof-of-concept allows one to compare the anatomy of females and males based on molecular markers (specific m/z distribution on spider slices). This is the first time this approach has been performed on a spider and could allow identifying regionalization on toxin production in venom glands when applied to other venomous animal groups. In the next year, this technology could also drive the identification of the spatial activity of toxin in tissue slice after envenomation by comparing the distribution of toxin in the tissue and the spatial distribution of substrate of some toxin/enzyme or again markers of inflammatory response and cellular death.

**Keywords:** MALDI imaging; *Steatoda nobilis*; venomics

### 6.19. Characterization of Innovative Animal Toxins for the Functional Study of Angiotensin Receptors

Anne-Cécile Van Baelen ^1,^*, Denis Servent ^1^, Nicolas Gilles ^1^, Philippe Robin ^1^

Université Paris-Saclay, CEA, Département Médicaments et Technologies pour la Santé (DMTS), SIMoS, F-91191 Gif-sur-Yvette, France.

* Correspondence: anne-cecile.vanbaelen@cea.fr

**Abstract:** An overactivation of the renin–angiotensin system (RAS) with an increase in angiotensin II level is the main cause of arterial hypertension. AT1 receptor antagonists are the goal standard for the treatment of this pathology. Nevertheless, other RAS-related GPCRs (AT2, Mas and MrgD) are also involved in the regulation of blood pressure but are under-exploited due to the lack of knowledge of their physiological properties. Furthermore, the development of biased ligand activating only certain intracellular signaling pathways is in full swing. The discovery of new ligands with original functions would be of great interest to better understand these receptors. Toxins from venomous animals are reticulated peptides that display antagonist, agonist or allosteric functions on GPCRs, and because of their high target specificity and selectivity, they constitute a large reservoir of unexploited promising ligands. The screening of an 800 toxin library was performed on the AT2 receptor by the mean of a radioligand binding assay, and it highlighted three original toxins which originate from different organisms with different structural profiles. These toxins display a micromolar affinity on AT2 and AT1 receptors, and so far, pharmacological trials are more advanced on this receptor. A-CTX-cMila is a small cyclic toxin of seven residues from the cone snail *Conus miliaris*, demonstrating a competitive antagonism on AT1 receptor with micromolar affinity, blocking both G-protein and β-arrestin pathways. A-CTX-MilVa, from the same cone snail, is composed of 20 residues and seems to be an AT1 agonist activating Gq protein pathway. Finally, A-TRTX-Por1a from the spider *Poecilotheria ornata*, is a three disulfide bridges peptide of 29 residues, which also appears to display an agonist activity on the AT1 receptor. These preliminary results describe the first animal toxins active on the RAS and highlight animal venoms as a prolific source of angiotensin receptor binders. A-CTX-cMila, A-CTX-MilVa and A-TRTX-Por1a, with their interesting structural and pharmacological profiles, may represent promising tools for the study of angiotensin receptors. More investigations are needed to characterize these toxins fully, especially on AT2. This work highlights a new family of GPCRs targeted by toxins.

**Keywords:** angiotensin; GPCR; toxin

### 6.20. Mast Cells Modulate the Immune Response and Redox Status of Intestinal Tissue in Induced Venom Pathogenesis

Nehla Zerarka-Chabane *, Fatima Laraba-Djebari, Djelila Hammoudi-Triki

University of Science and Technology Houari Boumediene (USTHB), Faculty of Biological Sciences, Laboratory of Cellular and Molecular Biology, Bab-Ezzouar, 16111 Algiers, Algeria.

* Correspondence: chabane.nehla88@gmail.com

**Abstract:** The inflammatory response caused by scorpion venoms components is a key event in the pathogenesis of scorpion envenomation. The binding of neurotoxins on their targets leads to a massive release of inflammatory mediators, such as cytokines, eicosanoids and bioactive amines from activated immune cells. Moreover, mast cells undergo maturation and polarization in gut mucosal surfaces and can alter intestinal permeability, an important factor in many inflammatory mucosal disorders. The c-Kit ligand, stem cell factor (SCF), is a major regulator of mast cell migration, development, growth, survival and activation. This study was conducted to investigate the effect of mast cells in modulating intestinal inflammation induced by scorpion venom. NMRI mice were injected by a sublethal dose of *Androctonus australis hector* (Aah) venom (0.5 mg/kg, subcutaneously) and sacrificed 24 h after envenomation; blood and organs were then collected for further analysis. The assessment of inflammatory response and the evaluation of redox status biomarkers were performed in the small intestine and colonic homogenates. Hematoxylin–eosin staining was used for general histological assessment, and toluidine blue staining was performed for identification and quantification of mast cells. The results revealed that Aah venom induced a significant increase in inflammatory cell infiltration, nitrite oxide levels and malondialdehyde (MDA) concomitantly with a significant decrease in catalase and glutathione S-transferase activities companied by intestinal tissue alteration. Furthermore, the percentage of mast cells was increased in the peritoneal cavity. Pharmacological inhibition of tyrosine kinase receptors alleviated these alterations. These findings could help to better understand the mechanisms involved in scorpion envenoming syndrome and develop potential drugs targeting mast cells for the management of inflammatory disorders of the gut.

**Keywords:** mast cell; redox status; scorpion venom

## Figures and Tables

**Table 1:** Comparison of the classification of proteins between *A. australis* and *A. mauritanicus*.

	*A. australis* (%)	*A. mauritanicus* (%)
Toxins	54.9	72.4
Non-toxins	16.9	12.3
Cellular components	26.1	13.5
Proteins not assigned	2.1	1.8

## Data Availability

Not applicable.

